# Cervical screening participation and access facilitators and barriers for people with intellectual disability: a systematic review and meta-analysis

**DOI:** 10.3389/fpsyt.2024.1379497

**Published:** 2024-07-26

**Authors:** Rosalie Power, Michael David, Iva Strnadová, Lauren Touyz, Caroline Basckin, Julie Loblinzk, Heather Jolly, Elizabeth Kennedy, Jane Ussher, Sally Sweeney, Ee-Lin Chang, Allison Carter, Deborah Bateson

**Affiliations:** ^1^ Translational Health Research Institute, Western Sydney University, Sydney, NSW, Australia; ^2^ The Daffodil Centre, University of Sydney, a joint venture with Cancer Council NSW, Sydney, NSW, Australia; ^3^ School of Medicine and Dentistry, Griffith University, Gold Coast, QLD, Australia; ^4^ Faculty of Arts, Design and Architecture, School of Education, University of New South Wales Sydney, Sydney, NSW, Australia; ^5^ Disability Innovation Institute, University of New South Wales, Sydney, NSW, Australia; ^6^ Self Advocacy Sydney, Sydney, NSW, Australia; ^7^ Family Planning Australia, Sydney, NSW, Australia; ^8^ Sexual Health and Reproductive Equity Research Group, UNSW Kirby Institute, Sydney, NSW, Australia

**Keywords:** cervical screening, intellectual disability, facilitators, barriers, cancer screening, early detection of cancer

## Abstract

**Background:**

The World Health Organisation’s vision of eliminating cervical cancer as a public health problem is achievable, but elimination must be achieved equitably, including for people with intellectual disability. A better understanding of cervical screening within the context of the lives of people with intellectual disability is needed. This study systematically reviewed research on the rates of cervical screening participation among people with intellectual disability, and facilitators and barriers that affect participation.

**Method:**

Six electronic databases were systematically searched: MEDLINE, CINAHL, Scopus, PsycINFO, Embase and Pro-Quest Central Social Sciences Collection. Empirical studies published between 1986 and 2023, in English language peer-reviewed journals were eligible for inclusion. Further articles were identified through forward and backward citation tracking, and hand-searching the index lists of two key journals. Two authors screened the studies, extracted the data and collated study outcomes using a standardised software program. A meta-analysis was performed using the DerSimonian and Laird method to estimate pooled effect sizes in prevalence rates and odds ratios (ORs). The socio-ecological model (SEM) was used as a framework to thematically analyse facilitators and barriers impacting participation in cervical screening.

**Results:**

Sixty-three articles met the inclusion criteria. Of these, 42 reported on rates of cervical screening participation and 24 reported on facilitators or barriers to cervical screening for people with intellectual disability. Overall, the studies reported a screening prevalence of 35% (95% CI: 26% to 45%), indicating that just over a third of people with intellectual disability have had cervical screening. The pooled odds ratio of 0.30 (95% CI: 0.23 to 0.41) indicated that people with intellectual disability are significantly less likely to have a cervical screening test compared with people without intellectual disability. Most studies examined individual and interpersonal factors impacting cervical screening. These included: (i) fear and anxiety among people with intellectual disability, (ii) misassumptions preventing screening participation, (iii) the role of support people, (iv) the need for education, (v) accessible information, and time to prepare for screening, (vi) patient-provider communication including challenges obtaining informed consent, and (vii) healthcare provider lack of confidence.

**Conclusion:**

Future research, policy and practice efforts must address barriers to cervical screening participation among people with intellectual disability and ensure these efforts are co-produced and community-led. This is critical to ensuring equity in global and local efforts to eliminate cervical cancer.

## 1 Introduction

Cervical cancer is a major public health problem and is the fourth leading cause of cancer incidence and death in women worldwide (1). However, cervical cancer can be prevented (2, 3), and it can be cured if detected at an early stage and treated effectively (3). The primary cause of cervical cancer is persistent infection with oncogenic types of the human papilloma virus (HPV) (4), a common viral infection of the genital tract, transmitted through sexual contact. The three pillars of prevention include HPV vaccination, cervical screening with a high-performance test, and treatment of pre-cancerous lesions (5). Countries with organised screening programs are increasingly switching from cytology-based screening with Pap smears to primary HPV screening (6). People with oncogenic HPV detected on their screening test can then either undergo surveillance with repeat testing or be referred for colposcopy and, if necessary, be treated for pre-cancerous lesions. Remarkable progress has been made in reducing cervical cancer-related diagnoses and mortality through these population-based screening programs, resulting in up to a 92% increase in survival rates (7). However, there are disparities in participation in cancer screening programs.

In 2020, the World Health Organisation (WHO) launched a Global Strategy to accelerate the elimination of cervical cancer as a public health problem (5). To be successful, elimination must be achieved equitably, including for cisgender women, trans and non-binary people with intellectual disability. Globally, 78 million people (1% of the population) have intellectual disability (8). This population are reported to have significantly poorer health and healthcare experiences compared with people without disability (9), including being twice as likely to die prematurely from preventable causes (10, 11) including cervical cancer (12) and six times more likely to experience barriers accessing healthcare services (13). Both the Disability (14) and Reproductive Justice Movements (15) have long recognised that structural and systemic issues, including negative social discourse about people with disability, impact access to healthcare and that multiple, intersecting forms of oppression deny people with disability the right to make decisions about their bodies (16). Barriers include lack of accessibility among services (including financial and transport issues) (13), communication issues (17), low levels of education including exclusion from sex education and information (18), and healthcare provider stigmatising (19) and discriminatory (20) attitudes and lack of knowledge (19).

People with intellectual disability are reported to have unique risk factors for cervical cancer, including low participation in HPV vaccination programs (21), and high rates of sexual abuse (22), including child sexual abuse (23), potentially increasing exposure to HPV. Despite the importance of cervical screening for this population, minimal attention has been paid to the barriers and facilitators of cervical screening. Currently, there are no internationally representative data on cervical screening participation among people with intellectual disability, and most countries lack screening data for this population group, except for those with nationally linked data sets such as Sweden (24). Broader disability research (i.e., focused on people with other forms of disability, including physical disability) has found that women with disability have 0.63 lower odds of participating in cervical screening (25), and are more likely to receive a later diagnosis of cervical cancer, less treatment, and to have higher mortality rates, compared with women without any disability (26). As findings are potentially worse for people with intellectual disability (11), there is a need to understand cervical screening participation rates for this group to inform prevention, policy and practice. There is also a need to understand facilitators and barriers that impact screening uptake, to enable long-term systemic change (15) for people with intellectual disability to equitably engage in cervical screening programs. To address these needs, our research questions were:

1. What percentage of people with intellectual disability participate in cervical screening?
2. What is the likelihood of people with intellectual disability participating in cervical screening compared with people without intellectual disability?
3. What are the facilitators and barriers that influence participation in cervical screening by people with intellectual disability?

An Easy Read version of this paper is available in the **Supplementary Material**.

## 2 Methods

This systematic review is part of ScreenEQUAL, a 3-year Australian Government National Health and Medical Research Council (NHMRC) funded multifaceted, inclusive study, using a co-production framework (27, 28), to improve cervical screening in people with intellectual disability. Our multidisciplinary team included social scientists, clinician researchers, non-government organisation (NGO) health promotion experts and includes an advisory group of people with intellectual disability and cervical screening healthcare providers.

This review was conducted according to a pre-defined protocol, registered with the International Prospective Register of Systematic Reviews (PROSPERO CRD42023393799). It includes a meta-analysis, which involves using statistical techniques to synthesise the data from several studies into a single quantitative estimate or summary effect size (29). The methods used for the review align with Preferred Reporting Items for Systematic Reviews and Meta-analyses (PRISMA) guidelines (30).

### 2.1 Inclusion criteria

Predefined inclusion criteria were articles that: (a) report rates, facilitators or barriers to cervical screening participation among people with intellectual disability. Articles were considered in scope if they defined their sample as being people with intellectual disability, learning disability, developmental disability/disorder or discussed a specific intellectual disability diagnosis such as Down Syndrome; (b) empirical research papers published in peer-reviewed English language journals; (c) published between 1986 to 2023. The year 1986 was selected to align with the development of the first WHO guidelines for cervical screening (31). Quantitative, qualitative and mixed-methods papers were included. Review articles, opinion pieces and grey literature were excluded. Studies were also excluded if they did not report specific results about cervical screening (i.e., if results about cervical screening could not be distinguished from results about other cancer screening programs) or if they did not report results specifically about participants with intellectual disability (i.e., if results of participants with intellectual disability could not be distinguished from the results of participants with other disabilities).

### 2.2 Search strategy

Six electronic databases, MEDLINE (OVID), Cumulative Index to Nursing and Allied Health (CINAHL) (EBSCO), Scopus (Elsevier), PsycINFO (EBSCO), Embase (OVID), ProQuest Central Social Sciences Collection (ProQuest), were searched between January and February 2023. The search strategy focused on terms and synonyms for intellectual disability (such as learning disability and developmental disorders), specific intellectual disability diagnoses (such as Down Syndrome) and cervical screening (such as Papanicolaou test, cervical smear, early detection of cancer) (**Supplementary Table 1**). Medical subject heading (MeSH) terms and free text terms were combined. Further articles were identified through forward and backward citation tracking of the included articles, and hand-searching the index lists of two key international peer-review journals that focus on intellectual disability research, Journal of Applied Research in Intellectual Disabilities and Journal of Intellectual Disability Research.

### 2.3 Study selection

**Figure 1:**
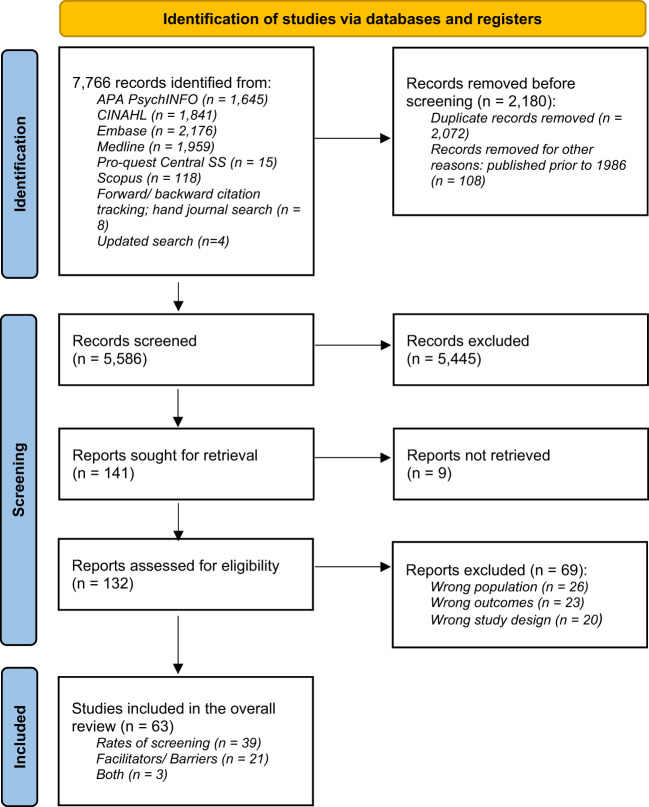
PRISMA chart.

Study selection is shown in **Figure 1**. A total of 7,762 records were identified from the searches and 2,180 duplicates were removed. Two reviewers (RP, LW) independently screened the title and abstract of articles for eligibility and removed a further 5,445 articles. The full text of the shortlisted articles was then examined against the eligibility criteria by the two reviewers resulting in a further 69 articles being excluded. Disagreements between reviewers were resolved through discussion with the senior author (DB) until a consensus was reached. Inter-rater reliability was calculated using Cohen’s kappa, with the average score being very high, **_K_** =0.96. The search strategy was re-run in August 2023 to identify studies published since the original search. A further four studies were identified, resulting in a total of 63 articles included in this review.

### 2.4 Critical appraisal of methodological quality

The Standard Quality Assessment Criteria for Evaluating Primary Research Papers From Various Fields (32) was used to assess the quality of each study. Two authors (RP, IS) independently reviewed each study and assigned scores of yes (2), partial (1), no (0) and N/A (1) for each quality assessment criteria (14 items for quantitative and 10 for qualitative studies). Disagreements between reviewers were resolved through discussion until a consensus was reached. A total score was then derived for each study, expressed a decimal between zero (lowest possible quality) and one (highest possible quality) indicating the strength of evidence and any concern of bias. For mixed methods studies, quantitative and qualitative scores were calculated, with the higher value used to define the study’s overall quality. A quality threshold of 0.55 was set to exclude the lowest quality bracket to ensure that questionable evidence and findings were not included (32). Interrater agreement for the quality assessment achieved a rating of “almost perfect” (33)^(p.3)^ using Cohen’s Linearly Weighted Kappa (κ) for each criterion in each study. This was calculated using Vassar Stats online calculator (34), which yielded a result of κ=0.9781 (observed kappa).

### 2.5 Data extraction and analysis

#### 2.5.1 Meta-analysis

This meta-analysis was performed by calculating pooled screening prevalence and odds ratios (ORs) for studies with comparators. For each, random effects modelling was applied to calculate effect sizes and 95% confidence intervals (95% CI) calculated using the DerSimonian and Laird method (35) and presented visually by forest plots. Prevalence rates were transformed using the Freeman-Tukey double arcsine method with the corresponding back-transformation equation (36, 37). For ORs, and when reported, the adjusted OR for screening participation from an individual study was included. In comparative studies where this was not the case, unadjusted ORs with corresponding standard errors were calculated. Statistical heterogeneity was quantified by the I-squared statistic and tested using Cochran’s Q statistic (38, 39). To visually and analytically investigate the source of heterogeneity, subgroup analysis and meta-regression were undertaken, respectively. In addition, for a time-cumulative meta-analyses, studies were arranged in chronological order, with multiple meta-analyses being performed by grouping studies by year. Publication bias, failure to publish the results of a study based on the direction or strength of the study findings, was assessed using Egger’s regression test (40). All statistical tests were two-sided, and a p-value of less than 0.05 was considered statistically significant. All analyses were performed using Stata Version 18.0 (Stata Corporation, College Station, TX, USA).

#### 2.5.2 Thematic analysis

Thematic analysis was conducted on the included data about facilitators or barriers to cervical screening for people with intellectual disability. The Socio-ecological Model (41) (SEM) was used as a guiding framework for this analysis. The model provides a multi-level lens for understanding facilitators and barriers to cervical screening as it recognises that health behaviours are shaped not only by an individual’s characteristics but also by social and environmental influences (41). Initially, data were extracted from the included studies by one author (EK), into a standardised software analysis program (Covidence) (42). A second author checked this for completeness and accuracy and disagreements were resolved through discussion (RP). A pre-defined data extraction template was used to organise information using the five levels of the SEM: (1) *individual*, such as attitudes and beliefs and sociodemographic characteristics; (2) *interpersonal*, such as healthcare provider communication and access to support; (3) *organisational*, such as service provider training, collaboration and accessibility; (4) *community*, such as promotion of, and education about, cervical screening; and, (5) *policy*, such as inclusion and exclusion in organised cervical cancer screening programs. Each level included the heading ‘other’, to allow recording of unanticipated facilitators or barriers. In reporting results, community and policy-level facilitators and barriers were presented together.

The extracted data were read and re-read to identify patterns, similarities, and differences across the studies. Data under each heading were summarised to facilitate the identification and development of themes, with the broader research team consulted on the interpretation and reporting of data. Different perspectives on the same data by the multi-disciplinary researchers helped the team reflect on and develop themes. This iterative process produced a textual description highlighting the common facilitators and barriers to cervical screening among individuals with intellectual disability. Analysing facilitators and barriers in the interconnected levels of the SEM enabled identification of knowledge and gaps in the existing research to develop recommendations for effective interventions and future research to optimise cervical screening in people with intellectual disability.

#### 2.5.3 Note on terminology

This review encompasses research conducted in various countries, where diverse terminology is used to refer to people with cognitive disabilities - including intellectual disability, learning disabilities, learning difficulties, and developmental disabilities. For instance, in Ireland, Australia, and New Zealand, the term “intellectual disability” is used, while in the United Kingdom, the terms “learning difficulties” or “learning disabilities” are used. Our team of authors consists of academic researchers from Australia, who have extensive experience in conducting inclusive research. After thorough discussions within our team and considering the cross-cultural nature of this article, we have decided to use the term ‘people with intellectual disability’ (43).

## 3 Results

### 3.1 Overview of studies

A total of 63 articles met the inclusion criteria, reporting on rates of cervical screening participation (*n*=39), facilitators or barriers to cervical screening (*n*=21) or both (*n*=3). Studies were published between 1994 to 2023 (no studies published between 1986 to 1994 were identified) and were all conducted in high-income countries (44), see **Table 1**. Over a quarter of studies (*n*=16, 25.4%) focused on cervical cancer screening only. The remainder examined cervical screening alongside other cancer screening programs (i.e., breast or bowel) or as part of broader healthcare research including health behaviour change interventions. Studies sought data from people with intellectual disability (*n*=15, 23.8%), family members (*n*=8, 12.7%), paid support workers (*n*=7, 11.7%) and health providers (*n*=8, 12.7%), or accessed data from medical records or data linkage (*n*=38, 60.3%). Standard Quality Assessment scores ranged from 0.75 to 1 (**Supplementary Table 2**).

**Table 1:** Study characteristics (all included studies *n*=63).

Study characteristics	*n studies* (%)
Continent	
Asia	7 (11.1%)
Europe	21 (33.3%)
North America	31 (49.2%)
Oceania	4 (6.3%)
Study focus	
Cancer screening	
*Cervical cancer screening only*	16 (25.4%)
*Cervical and breast cancer screening*	13 (20.6%)
*Multiple cancer screening (i.e., cervical, breast, bowel)*	7 (11.1%)
Healthcare service utilization/delivery	13 (20.6%)
Health status assessment	10 (15.9%)
Sexual and reproductive health	4 (6.3%)
Study design	
Quantitative	50 (79.4%)
Qualitative	9 (14.3%)
Mixed-methods	4 (6.3%)
Participant groups ^a^	
People with intellectual disability	15 (23.8%)
Family members	8 (12.7%)
Paid support workers	7 (11.7%)
Health providers	8 (12.7%)
Medical records/data linkage	38 (60.3%)

a multiple groups possible.

### 3.2 Meta-analysis: cervical screening participation among people with intellectual disability

#### 3.2.1 Cervical screening prevalence among people with intellectual disability

**Figure 2:**
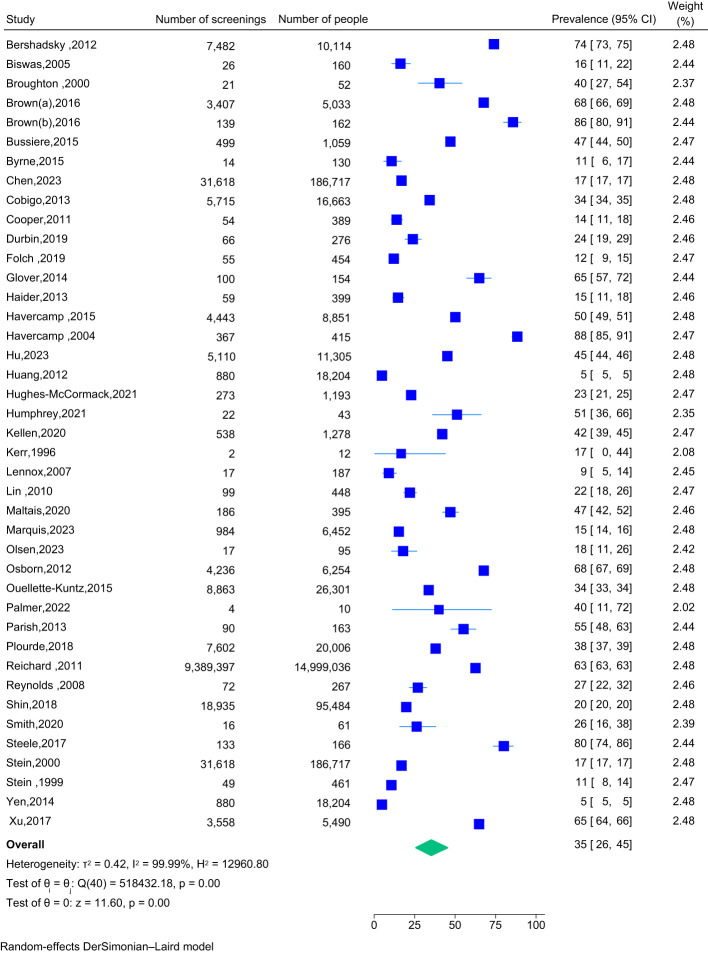
Forest plot for cervical screening prevalence for people with intellectual disability (*n*=41 studies).

Of the 42 studies included in the meta-analysis, 41 reported the prevalence of cervical screening among people with intellectual disability, with a total of 15,629,260 participants. Overall, the studies reported a screening prevalence of 35% (95% CI: 26% to 45%), indicating that just over one-third of eligible people with intellectual disability participated in cervical screening (see **Figure 2**). To assess heterogeneity (variability) among studies, a visual subgroup analysis by Year of Publication and Continent was performed (**Supplementary Figures 1**, **2**). For the Year of Publication, the only pairing displaying a noticeable difference was the comparison between studies published from 1996 to 2002 (Screening Prevalence: 18%; 95% CI: 11% - 26%) and studies published from 2017 to 2023 (34%; 28% - 40%). When estimates were pooled across four continents, noticeable differences were also seen between studies published in North America (53%; 44% - 62%) and the three other continents (Asia, Europe and Oceania). A multivariable meta-regression (**Supplementary Table 3**) identified significantly higher rates of screening in North America than the other continents (p<0.01), thereby confirming Continent to be a source of study heterogeneity. However, the Year of Publication was not seen to be a source of study heterogeneity, as its effect was not statistically significant (p=0.31), Additionally, there was no evidence of publication bias, with Egger’s test being non-significant (p=0.70).

#### 3.2.2 Pooled odds ratio of cervical screening among people with intellectual disability, compared to people without intellectual disability

**Figure 3:**
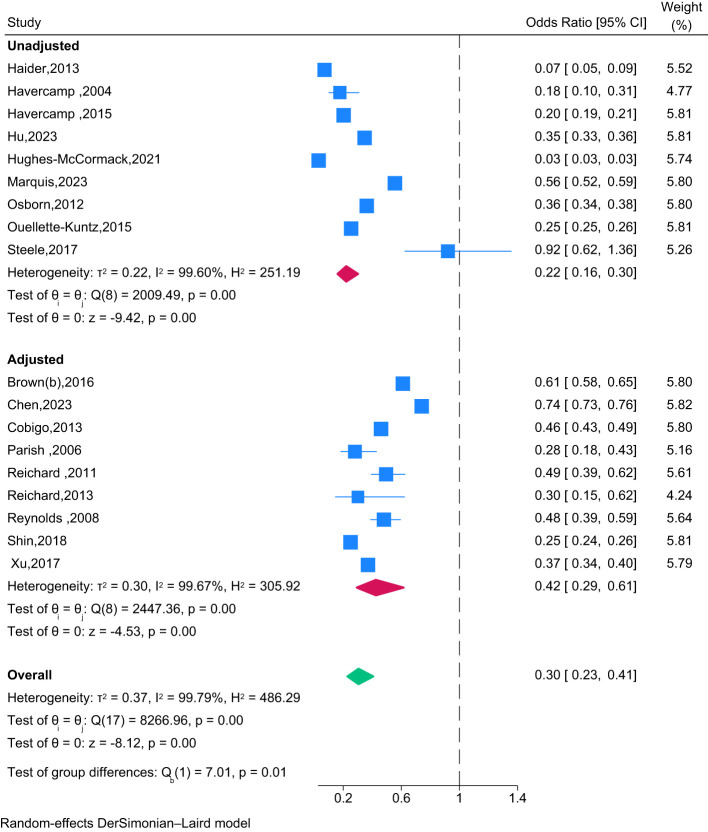
Forest plot of the association between screening participation and intellectual disability compared to those without intellectual disability by type of analysis (unadjusted, adjusted) (*n*=18 studies).

**Figure 4:**
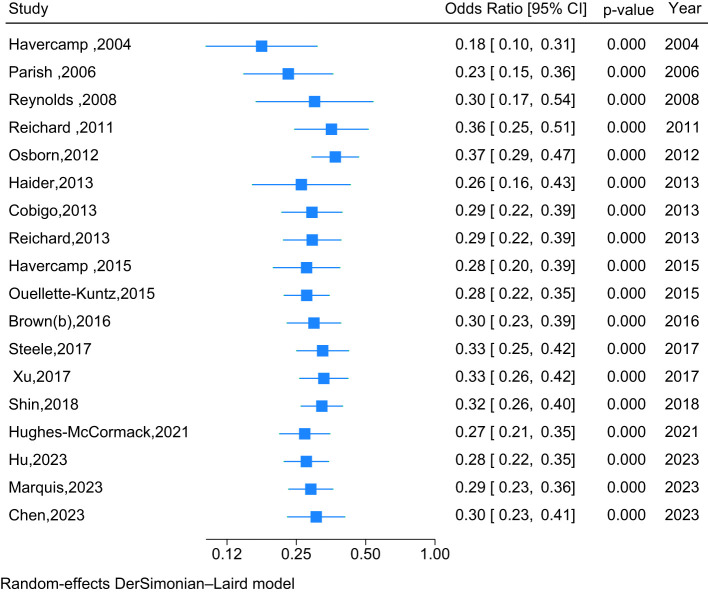
Cumulative forest plot of the association between screening participation and intellectual disability compared to those without intellectual disability, by year of publication (2004 – 2023).

Eighteen studies used a randomised controlled trial design with an appropriate control group to compare cervical screening participation of people with intellectual disability with participation by those without intellectual disability. The overall pooled OR of 0.30 (95% CI: 0.23 to 0.41) indicates that people with intellectual disability are significantly less likely to participate in cervical screening, compared with people without intellectual disability (**Figure 3**). Furthermore, whilst a time-cumulative forest plot (**Figure 4**) shows an upward trend in ORs from 0.18 (0.10 - 0.31) in 2004 to 0.29 (0.22 - 0.39) in 2013, over the past 10 years, the pooled OR has plateaued, increasing minimally to 0.30 (0.23 - 0.41). An analytical assessment of heterogeneity across Type of Analysis, Continent and Year of Publication by a multivariable meta-regression (**Supplementary Table 4**) confirmed the effects of Type of Analysis (p=0.45) and Year of Publication (p=0.97) as being non-significant. Whilst Oceania studies reported much lower screening rates for people with intellectual disability compared to studies from North America (*β*=-1.49; 95%CI: -3.37 - 0.40), the overall effect for Continent was also not found to be statistically significant (p=0.42). There was no evidence of publication bias, with Egger’s test being non-significant (p=0.68).

### 3.3 Factors impacting participation in cervical screening among people with intellectual disability

**Figure 5:**
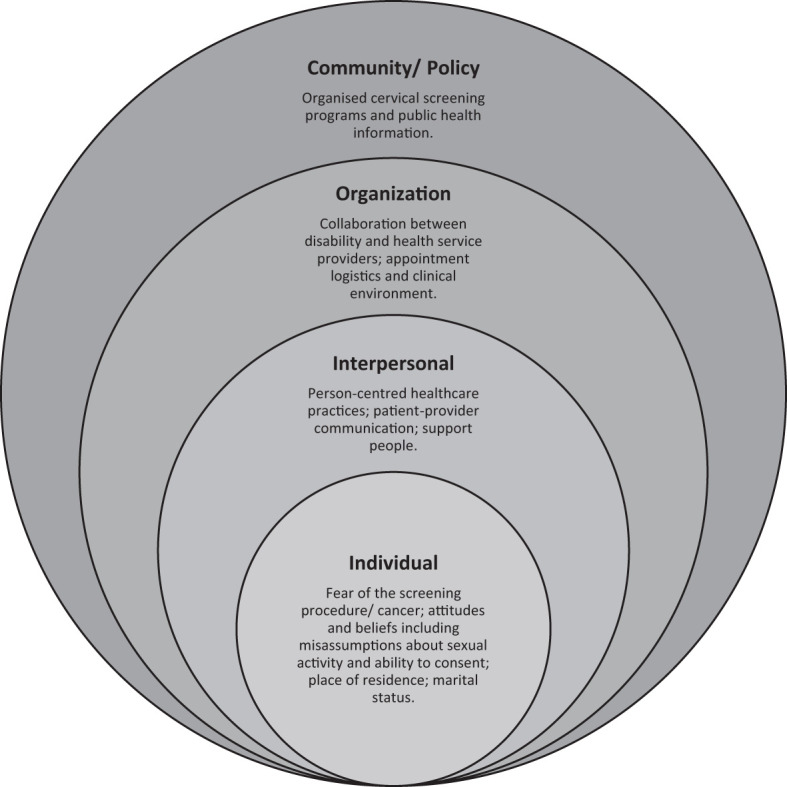
Socio-ecological factors impacting cervical screening participation for people with intellectual disability.

Twenty-four studies examined facilitators or barriers to cervical screening among people with intellectual disability. Included studies used either a qualitative (n=8), quantitative (n=11) or mixed methods approach (n=5). **Figure 5** presents facilitators and barriers to cervical screening, grouped by individual, interpersonal, organisational, community and policy-level factors from the Socio-ecological Model (SEM) and **Table 2** provides a summary of each of the studies. In the analysis below, we describe how the identified factors were understood to impact cervical screening participation for people with intellectual disability.

**Table 2:** Studies reporting barriers or facilitators to cervical screening (*n*=24).

Author	Year	Country	Research question/objective	Focus population/s and data collection method	Major findings
Agaronnik, Pendo (45)	2020	United States of America	To explore perceptions of providing reproductive health services to women with intellectual disability.	Individual interviews with physicians (n=20) across selected specialties (primary care, rheumatology, neurology, obstetrics/gynaecology, and orthopaedics). Three focus groups with rural and non-rural primary care physicians (n=22) across selected specialty (rheumatology, neurology, obstetrics/gynaecology, orthopaedics, and ophthalmology).	Physicians indicated that intellectual disability posed challenges to providing sexual and reproductive healthcare, including cervical screening. Physicians believed that cervical screening was not necessary for all women with intellectual disability; women with intellectual disability were believed generally not to be sexually active. Physicians reported time constraints and a lack of preparedness for performing screening procedures with women with intellectual disability.
Armin, Williamson (46)	2022	United States of America	Influences on breast and cervical cancer screening for Native American women with intellectual (or developmental) disability.	Semi-structured in-depth interviews with: - Women with intellectual (and developmental) disability (*n*=12). - Caregivers (*n*=12). - Healthcare or disability service providers (*n*=22). - Community members/leaders (*n*=2).	Barriers to screening were: Individual – financial concerns; individual attitudes about healthcare; fears about cancer or cancer screening; need for information about screening; general lack of health education; and beliefs about screening. Interpersonal – provider and caregiver bias; the importance of communication; family and caregiver relationships; and relationship with the healthcare provider. Community/institutional – distrust of allopathic medicine; preference for traditional medicine; traditional ways of knowing; limitations to receiving community services; and accessibility of healthcare facilities.
Breau, Thorne (47)	2020	Canada	How primary care providers and trainees recommend cancer screening to their patients with intellectual disability.	Survey and vignettes of fictional patients with family physicians (n=58), family medicine trainees (n=28) and primary care nurse practitioner students (n=9)	Negative attitudes towards community inclusion of people with intellectual disability predicted participants’ likelihood of recommending screening to fictional patients.
Breau, Thorne (48)	2023	Canada	Experiences of primary care providers in recommending cancer screening to their patients with intellectual disability.	Interviews with family physicians (*n*=2) and family medicine trainees (*n*=10).	Intellectual disability was reported to be an unacceptable reason to withhold preventive care such as cervical screening.
Broughton and Thomson (49)	2000	England	Views and experiences of women with intellectual (learning) disability of the cervical smear test.	Interviews with women with intellectual (learning) disability (*n*=52) and their carers (*n*=34).	Factors enabling women with intellectual (learning) disability to have a cervical smear test included preparation work to build understanding about the test; adequate communication; support from carers; and the need for health centre staff to understand women’s needs. Women with intellectual (learning) disability reported anxiety about the test, including concern about pain. Carers reported being told by medical professionals that cervical screening was not necessary for women with intellectual (learning) disability, unless the women were sexually active.
Conder, Mirfin-Veitch (50)	2018	New Zealand	(1) To explore the knowledge and experiences of women with intellectual disability of breast and cervical screening. (2) To identify factors that health practitioners and disability service providers perceived as facilitating or impeding the participation of women with intellectual disability in generic health screening.	Semi-structured interviews with disability and health professionals (*n*=5).	Influences on screening participation among women with intellectual disability were: perceptions of women as physically or behaviourally capable of participating in screening; perception of women as sexually active; having a pathway onto the screening program (i.e., annual health checks); disability service provider knowledge of women’s screening status; perceived ability of women to provide informed consent. Healthcare and disability service provider strategies to provide women with information about screening included: allowing time for consideration and consent; choosing how to relay information; ensuring the woman was informed of the results of her screen.
Fortney and Tasse (51)	2021	United States of America	The effects of rurality on access to preventative healthcare and services and health status.	National Core Indicators Adult Consumer Survey 2015-2016 Final Report (*n*=17,682 people with intellectual (and developmental) disability	Women with intellectual (and developmental) disability living in non-metropolitan areas were less likely (*p*<0.001) to receive a pap test than women with intellectual (and developmental) disability living in metropolitan areas.
Langan, Whitfield (52)	1994	England	To explore the role of carers, paid and unpaid, with regard to the primary healthcare of people with intellectual (learning) disability, and information about GP care from the perspective of carers.	Interviews with carers (*n*=76) of 81 individuals with intellectual (learning) disability.	Cervical screening was not provided to women with intellectual disability because: cervical screening was overlooked; perceived as being too uncomfortable; not considered necessary because of presumed sexual inactivity; or because the carer refused to give consent for the test.
Lin, Lin (53)	2011	Taiwan	To describe caregiver attitudes and to examine determinants of gynaecological health for women with intellectual disability.	Questionnaire with paid caregivers of women with intellectual disability (*n*=1,152).	94.4% of caregivers agreed that health check-ups for women with intellectual disability should include a pap smear.
Lin, Sung (54)	2010	Taiwan	To explore the perceptions and experience of primary care physicians in the pap smear screening provision for people with intellectual disability.	Survey with primary care physicians (*n*=168).	51.5% of services had practical experience conducting pap smears for women with intellectual disability. 90% of primary care physicians said women with intellectual disability need regular pap smears.
Lloyd and Coulson (55)	2014	England	Intellectual (learning) disability nurses’ experiences of supporting women with intellectual disability to access cervical screening.	Semi-structured interviews with intellectual (learning) disability nurses (*n*=10).	Barriers to cervical screening included: limited health literacy, negative attitudes and beliefs and competing demands. Primary care professional barriers included time pressures, limited exposure to people with intellectual disability and lack of appropriate knowledge, attitudes and skills. Attendance at cervical screening was facilitated by prolonged preparation work undertaken by intellectual (learning) disability nurses, helpful clinical behaviours in the primary care context and effective joint working.
Parish, Moss (56)	2008	United States of America	To understand the perspectives of individuals with intellectual (developmental) disability about their health status, health promotion behaviours, and healthcare services they receive.	Seven focus groups with people with intellectual (developmental) disability (*n*=30; female=12, 40%).	Women with intellectual disability were reported to be too frightened to receive the Pap test regularly.
Parish, Rose (57)	2011	United States of America	To test the *Women Be Healthy* intervention, designed to improve women’s knowledge of cervical and breast cancer screening.	Randomised control trial with women with intellectual (developmental) disability (n=175) to have cervical and breast screening. Experimental group – weekly health education program, control group regular vocational training or educational activities.	Changes in women’s knowledge about cervical screening were non-significant.
Parish, Swaine (58)	2012	United States of America	To examine the extent of women’s knowledge about cervical and breast cancer screening, to inform the *Women Be Healthy* intervention.	Interviews with community-dwelling women with intellectual (developmental) disability (n=202).	Women with intellectual (developmental) disability had little knowledge of cervical screening: 35% knew the definition of a pap test; 19% knew the recommended pap test frequency; 55% correctly identified the instrument used for a Pap test; and 41% correctly identified ways to decrease anxiety related to pelvic exams. Women living with a spouse had greater knowledge than those living with family caregivers or in residential settings.
Parish, Swaine (59)	2013	United States of America	To examine receipt of cervical cancer screening and determinants of screening for women with intellectual disability.	Medical records from 2006-2010 of community-dwelling women with intellectual disability in community dwellings (*n*=163).	Women with intellectual disability who lived in residential facilities, those who lived in rural communities, and those who had an obstetrician gynaecologist had higher rates of cervical screening than other women with intellectual disability.
Plourde, Brown (60)	2018	Canada	To examine the association between the level of primary care continuity and breast and cervical cancer screening rates in women with intellectual disability.	Data were obtained from the Institute for Clinical Evaluation Sciences (healthcare data) and the Ontario Ministry of Community and Social Services (social assistance and disability support program).	Women with high and moderate continuity of care were less likely to have a pap test than women with low continuity of care.
Rees (61)	2011	England	To estimate the awareness of cancer screening awareness among frontline staff working with people with intellectual (learning) disability.	Questionnaire with doctors (n=5), community intellectual (learning) disability nurses (n=20), and health facilitation nurses (n=7).	Knowledge of cervical screening was poor. 94% of participants were unable to correctly identify the age eligibility age for smear test; 28% provided out-of-date information.
Son, Parish (62)	2013	United States of America	To examine the concordance between self-reported and medical record data of cervical and breast screening for women with intellectual disability.	Face-to-face interviews and medical records from women with intellectual disability (*n*=155).	Participants overreported their receipt of pap tests.
Stein (63)	2000	England	To examine general practitioners’ beliefs about caring for people with intellectual (learning) disability.	Questionnaire with general practitioners (*n*=48).	54% of participants agreed they would offer a cervical smear to a woman aged between 20 and 64 with intellectual (learning) disability, who had not had a cervical smear taking in the past, after suitable discussion.
Swaine, Dababnah (64)	2013	United States of America of America	To assess barriers to cervical and breast cancer screening from the perspective of female familial caregivers.	Semi-structured interviews with familial caregivers (*n*=32).	Reasons women with intellectual disability did not receive pap smear: not sexually active (57%), hysterectomy (35%), women with intellectual disability are uncomfortable with exam (14%), caregiver does not consent to exam (7%), Medicaid does not cover (7%). Reasons women with intellectual disability were comfortable with exams: caregiver explained procedure (75%), doctor explained procedure (25%), female physician (13%). Reasons women with intellectual disability were not comfortable with exam: embarrassment or shyness (56%), not sexually active/uncomfortable being touched (25%), women with intellectual disability believes exam is painful (19%), caregiver is not comfortable with exam (6%). Methods to make it easier for women with intellectual disability to have pap smear: caregiver explains procedure and remains in the exam room (64%), anti-anxiety medication (18%), women with intellectual disability familiar/comfortable with doctor (18%), female physician (9%), physician aware women not sexually active (9%).
Swaine, Parish (65)	2014	United States of America	To test a modified version of Women Be Healthy, Women Be Healthy 2, and compare its effectiveness in increasing knowledge gains to the original intervention.	Baseline and post-intervention interviews with women with intellectual disability: - Treatment (*n*=98) - Delayed treatment (*n*=35). - No intervention (*n*=65)	Women who received *Women Be Healthy 2* had increased knowledge overall compared with the women receiving no intervention.
Sykes, McGeechan (66)	2022	England	To understand knowledge of, attitudes towards and decision-making around cervical and breast cancer screening in women with intellectual (learning) disability, family carers and paid carers.	Q methodology (to systematically study persons’ beliefs and attitudes) with women with intellectual (learning) disability (*n*=13), family carers (*n*=3), and paid care workers (*n*=5)	Women with intellectual (learning) disability should have ownership over their healthcare decisions, including whether to attend cervical screening or not. Family carers and paid workers were protective in supporting women with intellectual (learning) disability who decided to attend cervical screening.
Wicks (67)	2007	United Kingdom	To explore differences between screening users and non-users in terms of where they attribute the source of power in maintaining their health and in their knowledge of cervical screening.	Questionnaire with open-ended response options with women with intellectual (learning) disability (*n*=19).	Many women with intellectual (learning) disability were aware of, information about, and managed to participate in cervical screening. Women who participated in screening were more knowledgeable and considered the test more valuable than non-users.
Wood and Douglas (68)	2007	United Kingdom	To evaluate primary care professionals’ views and current practices in providing cervical screening to women with intellectual (learning) disability.	Questionnaire and interviews with GPs (*n*=10), practice nurses (*n*=2), practice managers (*n*=6) and members or administrative staff (*n*=2).	Health services lacked robust mechanisms to identify patients with intellectual (learning) disability. Health professionals made pragmatic decisions when considering screening for women unable to give informed consent, guided by the presence or absence of behavioural consent.

#### 3.3.1 Individual level facilitators and barriers

##### 3.3.1.1 Fear and anxiety are barriers to cervical screening for people with intellectual disability.

Numerous studies (46, 49, 52, 55, 56, 64, 67) reported fear and anxiety among people with intellectual disability, regarding cervical screening, with many “too frightened” (56)^(p.419)^ to receive the test. In studies with people who had previously experienced cervical screening, participants used words such as “painful” (49)^(p.908)^ (67)^(p.14)^, “awkward” (49)^(p.908)^ and “scary” (67)^(p.14)^ to describe the test. In one study, a participant with intellectual disability explained, ‘‘I hate Pap smears. They pinch when they are inside of you. I kick and scream when I have to get them.’’ (56)^(p.419)^.

Studies with family members found that women with intellectual disability were often uncomfortable with the screening procedure, due to “general embarrassment or shyness with the private nature of the exam” (64)^(p.68)^ and “because of the women’s lack of sexual experience.” (64)^(p.66)^ Family members also suggested that women with intellectual disability may require anti-anxiety medications or sedatives to tolerate the exam including the use of a speculum (64). Other study participants, including disability and healthcare providers, acknowledged that histories of sexual abuse among people with intellectual disability could be a barrier to screening participation (46).

Research participants frequently reported that people with intellectual disability experienced negative experiences with healthcare providers, such as not being listened to and respected, which impacted their engagement with cervical screening, as explained by an intellectual disability nurse:

[ … ] she [the practice nurse] attempted to do the smear test, the lady couldn’t cope with it and was absolutely screaming and what have you and I just said ‘stop’. I don’t know why but the practice nurse didn’t stop at that point she just tried to carry on. So I intervened and said ‘stop’ so she did and then she was really sort of aggressive to me and mum saying ‘why have you come for this appointment, why have you put this woman through this?’ and tried to blame us. (55)^(p. 138)^.

One study (46) reported that women with intellectual disability might avoid cervical screening due to fear that they could be diagnosed with cancer and that treatment would reduce their ability to live independently.

##### 3.3.1.2 Misassumptions preventing cervical screening participation

Most studies (45, 46, 48–50, 52, 55, 60, 61, 63, 64) reported that family members and disability and healthcare providers held misassumptions about the need for people with intellectual disability to have a cervical screening test. Incorrect beliefs included that people with intellectual disability were sexually inactive, precluding cervical screening. For example, in one study, only 54% of doctors said they would offer cervical screening to “a woman aged between 20 and 64 with intellectual disability” who “had not had a cervical smear test taken in the past [ … ], after suitable discussion” (63)^(p.13)^, with most citing sexual inactivity as a principal determinant of their decision. A family member in another study explained, “I do feel that because a person has some sort of intellectual difficulty their sexual life is dismissed. They should have the same screening as any person has.” (52)^(p.361)^ Only one study (46) reported awareness among service providers regarding the high rates of sexual assault experienced by people with intellectual disability. It emphasised the importance of avoiding assumptions about HPV exposure when determining cervical screening eligibility:

Women with disability are some of the highest risk for unwanted sexual assault. And so you can’t assume, you know, that somebody hasn’t been exposed to HPV even if it wouldn’t seem as if they would have had an opportunity to be exposed. (46)^(p.8)^.

Health care providers reportedly made presumptions about the healthcare priorities of patients with intellectual disability such as that the patient’s primary diagnosis presented more pressing need than cervical screening. In one study, a rural woman with intellectual disability stated, “she’s [healthcare provider] more concerned with my disability than anything.” (46) In the same study a disability service provider commented:

For people with disabilities in general, they tend not to get asked about healthcare screening and [are] more likely to be asked about stress and stuff like that. So, women particularly with disability are often assumed not to be sexually active, they never get a sexual history taken. It’s called diagnostic overshadowing … where that primary diagnosis is all the physician can see. (46)^(p.5)^.

Another study found that whilst many healthcare providers said they would “never take a unilateral decision not to invite women with intellectual disability for cervical screening,” (68) ^(p.88)^ some providers indicated that “women with severe intellectual disability would only ‘sometimes’ be invited”^(p.88)^. Health care provider misassumptions regarding the importance of cervical screening for patients with intellectual disability and their inconsistencies offering cervical screening for this group, left people with intellectual disability feeling “dismissed, insignificant and overlooked.” (46)^(p.6)^.

##### 3.3.1.3 Sociodemographic and other characteristics associated with low cervical screening participation

Several sociodemographic characteristics were reported to be associated with low participation in cervical screening for people with intellectual disability. Two studies (59, 69) reported that people with intellectual disability living with parents or relatives were significantly less likely to participate in cervical screening than those living in residential facilities as well as those living with parents or relatives being quoted to have “alarmingly limited accurate knowledge”. (59)^(p.84)^ Studies reported mixed findings regarding access to cervical screening by location. One study (51) found that people with intellectual disability living in rural locations were significantly less likely to receive cervical screening compared with people with intellectual disability living in metropolitan locations. Conversely, another study (59) found that people with intellectual disability living in rural areas had a significantly higher likelihood of receiving a Pap test than those living in urban settings. People with intellectual disability who were married or who had tubal ligation surgery were reportedly more likely to have had a cervical screening test compared with people with intellectual disability who were not married or who had not had tubal ligation surgery (54). Other factors reported to be associated with cervical screening included sexual activity; number of sexual partners; pregnancy; and a past history of smoking (49).

#### 3.3.2 Interpersonal level facilitators and barriers

##### 3.3.2.1 “The right rapport, the right demeanour”: *The right* support people helped people with intellectual disability to participate in cervical screening

Several studies (46, 48, 49, 64, 66) reported that having a support person present (i.e., family members or support worker) was beneficial for people with intellectual disability when undertaking a cervical screening test. People with intellectual disability recommended taking a support person to cervical screening appointments because “having someone with you gives you more confidence.” (49)^(p.909)^ In one study, family members described providing comfort, such as “holding the woman’s hand” (64)^(p.69)^ and “repeating the phrase ‘breathe’ just to keep her calm.” (64)^(p.68)^ Overall, support people highlighted that having good rapport with the person with intellectual disability was crucial for empowering this group to participate in screening and to have a positive experience with the test. A disability service provider explained the factors they considered when selecting staff members to accompany a person with intellectual disability to a cervical screening appointment:

It’s about who has the right rapport, the right demeanour that suits the person … who they feel comfortable with [ … ] There’s no point putting someone on that’s been employed for two weeks to go and take someone … To become familiar with the person … may take up to a 12-month period … especially if you are from a culture where … women’s health screening is not talked about. (50)^(p.92)^.

While most studies reported positive perceptions of support people, one study noted that the “viewpoints [of caregivers] must be considered during the clinical encounter”, suggesting that “the presence of an additional caregiver may make coordination of care more burdensome and thus more difficult to offer cancer screening.” (48) ^(p.256)^ Overall, these findings provide important insight into the role of support people in the provision of cervical screening with people with intellectual disability.

##### 3.3.2.2 “I don’t go straight to the Pap smear first thing:” person-centred healthcare provider practices supporting cervical screening

Numerous studies (45, 46, 48, 55) provided information about strategies and approaches utilised by healthcare providers to successfully “[ … ] prepare women [with intellectual disability] psychologically for screening to enhance understanding, increase predictability and minimise anxiety.” (55) ^(p.143)^ Health care providers in these studies said they took time to explain the cervical screening procedure in depth, before the test was performed, including using “visual communication with pictures and diagrams.” (46) ^(p.7)^ One healthcare provider said, “I will draw it out, I explain what I’m doing before we do it” (46) ^(p.7)^ and another said, “we just have a uterus, not a picture, but a 3D little thing, where you can actually see and hold it towards my belly and say, ‘that’s where it is…’.” (45) ^(p.368)^ Health care providers in these studies said that they “don’t go straight to the Pap smear first thing” (46) ^(p.8)^, as an intellectual disability nurse explained,

[ … ] we go down just to look at the room, let them sit on the couch you know look at the speculum all those kind of things, you know the little brush that actually takes the specimen you know takes the cells away, just so they’ve got an understanding of what it involves. You know getting in position without doing anything invasive at the time and just maybe build on that so you take two or three trips maybe beforehand just to kind of desensitise and build that bit of insight really and obviously the person who’s going to actually do the procedure get them involved as well if possible [ … ] I would look at doing that really because that’s automatically going to make the person hopefully feel comfortable (55). ^(p.135)^.

Health care providers discussed adjusting their clinical practices to “meet them [patients with intellectual disability] where they’re at and not push them out of their comfort zone” (46) ^(p.8)^. For example, a healthcare provider said:

I’ve had a couple patients where they don’t want me to elevate the table up or they get really dizzy or anxious with that, so I have to leave the table basically pretty low to the ground, and then we still try to put their feet in the foot rests, but I kind of have to crouch on the ground sometimes, because I can’t raise the table up” (46) ^(p.8)^.

These person-centred strategies were reported to build trust between patients with intellectual disability and healthcare providers and build the knowledge of patients so that “[ … ] eventually you can get to the point where sometimes you can do a Pap smear.” (46) ^(p.8)^ However, a number of studies (45, 48, 50, 55) reported that clinical time constraints meant healthcare providers lacked the time to adequately explain the need and nature of cervical screening and perform the test. In these instances, healthcare providers were reported to ask patients to return for a second appointment to conduct the screening procedure. However, this was acknowledged to increase the risk of losing patients to follow-up.

##### 3.3.2.3 Challenges in conducting the cervical screening test: health care provider continuity, communication and consent

The importance for people with intellectual disability to feel comfortable with their doctor to have cervical screening, was discussed in a number of studies (46, 49, 60). A woman with intellectual disability said that she could proceed with having a cervical screening test because, “I’m able to talk with my doctor. I’m able to explain to my doctors what’s wrong with me.” (46) ^(p.8)^ In another study, a person with intellectual disability said they found it helpful when “the nurse tried to relax me, talked with me and joked with me to take my mind off it.” (49) ^(p.909)^ Although a positive relationship with the healthcare provider was important, in one study (60) people with intellectual disability were found less likely to receive cervical screening when they had a long-term relationship with their doctor, attributed to entrenched misassumptions by the doctor about sexual activity/inactivity and vulnerability to sexual assault (60). One study (46) reported that people with intellectual disability were more comfortable having a cervical screening test with female healthcare providers.

Studies (46, 49, 50, 55, 68) also reported healthcare provider concerns about navigating informed consent, described as a “long and repetitive process that has to be individually tailored to each woman.” (50) ^(p.91)^ Health care providers were reported to lack the communicative competence required to navigate consent and perform cervical screening with patients with intellectual disability, as an intellectual disability nurse commented:

[ … ] They [GPs] wouldn’t show any accessible information, they wouldn’t check for retention [of information], they wouldn’t even necessarily get the decision-making process right (55) ^(p.139)^.

Similarly, Conder, Mirfin-Veitch (50) reported that healthcare providers often directed questions to support people (i.e., family members or disability service providers) rather than the individual with intellectual disability, which could fail to elicit a full sexual history, including experiences of sexual abuse. Family members and disability and healthcare providers were also reported to believe that patients with intellectual disability could not understand information about cervical screening, as a service provider explained:

“…sometimes I feel like in certain situations, some facilities just think that because they’re not capable of understanding these things, they don’t take the time to explain it to them, so they automatically write them off as they don’t need it or they waive their right…” (46) ^(p.7)^.

In another study a healthcare provider commented, “I’ll talk to my patients, even the ones that aren’t able to interact at all, and I’ve had family members tell me ‘no they don’t understand you,’ but I’ll talk to them anyway.” (45) ^(p.367)^.

##### 3.3.2.4 Healthcare provider experience, skill and confidence

In several studies (46, 49, 50, 55, 59, 68), healthcare providers were reported to have a “lack of experience or skill or confidence” (55) ^(p.138)^ conducting cervical screening with people with intellectual disability. One study reported that only 57.5% of healthcare providers were confident in providing cervical screening for people with intellectual disability (54). An intellectual disability nurse explained:

[ … ] I don’t think we always realise how difficult it is for people who haven’t worked with people with an intellectual disability. We go in all guns blazing about reasonable adjustments but people who have no experience of people with an intellectual disability, it’s understandable sometimes why their decision-making process isn’t okay or maybe their approach isn’t. [ … ] I do think that we do have to think that someone’s experience of intellectual disability is very limited and you’re doing a very invasive procedure with someone who’s very distressed. You may not handle it that well (55). ^(p.138)^.

Some studies reported perceived healthcare provider difficulties in performing the test with “women who might find it difficult to keep still throughout the procedure.” (50) ^(p.89)^ For example, healthcare providers said they would not perform cervical screening for people with intellectual disability “where the level of disability is too high” as “it is such a very delicate procedure and a very delicate part of the human anatomy, and we felt that we couldn’t control the situation enough to do it properly.” (45) ^(p.368)^ Some doctors said they would refer patients with intellectual disability to a gynaecologist rather than performing these themselves (45). These findings were similar to another study (59) that found women who were regularly seeing a specialist obstetrician/gynaecologist were significantly more likely to receive cervical screening than those who were seeing a GP.

#### 3.3.3 Organisational level facilitators and barriers

##### 3.3.3.1 Collaboration between disability service providers and healthcare providers facilitates cervical screening

Facilitating positive experiences of cervical screening extended beyond the test itself and included the whole clinical experience, such as time spent in the clinic waiting room and “setting appointment times that worked for the woman, which might mean scheduling a longer appointment, or one at a particular time of day.” (50) ^(p.92)^ Studies reported that collaboration between disability and health services was essential to facilitate appointment logistics that worked for the person with intellectual disability. This included sharing “information from support services about the woman’s special needs [ … ] such as the need for a quiet waiting room [ … ] and [ … ] if the practice staff know there is going to be a delay, they actually ring if we need to know.” (50) ^(p.92)^ These person-centred approaches were reported to help alleviate anxiety and contributed to positive experiences of cervical screening. However, another study (50) reported that disability service providers often didn’t know the screening status of their clients, particularly if clients lived independently, making it difficult to facilitate participation in cervical screening.

Studies reported lack of clinical practices to ensure the cervical screening needs of patients with intellectual disability were met. This included that health services “rarely proactively consider the health needs of people with intellectual disability as a specific patient group with additional needs.” (68) ^(p.88)^.

Only one practice had nominated a member of staff to take lead responsibility for patients with disabilities (including intellectual disability), very few practices said they routinely kept up-to-date records of the named Community Intellectual Disability Team (CLDT) nurse for their patients under the Team’s care, awareness of the general health checks provided by the CLDTs was low, and practices rarely provided general health checks for patients with intellectual disability themselves (68)^(p.88)^.

In one study, a service provider recommended clinics adopt design features to improve women’s experiences of cervical screening, such as “making one room that’s colorful” and “making your rooms more friendly looking, bright.” (46) ^(p.10)^ These findings provide insight into optimising the roles of disability and health services in supporting access to cervical screening for people with intellectual disability.

#### 3.3.4 Community and policy level facilitators and barriers

##### 3.3.4.1 Exclusion of people with intellectual disability from cervical screening information

People with intellectual disability were reported to lack knowledge about cervical screening (46, 67), often believing they were not eligible for the test. A woman with intellectual disability stated, “I thought I wouldn’t be able to have a smear test. Women with intellectual disability feel like they can’t have a test, not like other women.” (67) ^(p.14)^ In one study (58) only 35% of participants with intellectual disability knew the definition of a Pap test and only 19% knew the recommended Pap test frequency. Studies attributed this lack of knowledge to the exclusion of people with intellectual disability from cervical screening information, as a service provider commented:

There’s a whole network of informal ways that people learn … and this goes across the board with a lot of health messaging, less accessible to people with disability, or they don’t see it as pertaining to them because it’s never targeted (46)^(p.6)^.

Standard cervical screening invitations and reminder letters often failed to meet the literacy needs of people with intellectual disability (55, 68). For example, in one study, only a minority of clinics surveyed “would always check whether a woman had an intellectual disability or other communication difficulty before issuing her invitation for cervical screening” (68) ^(p.89)^. The study reported that “even when issuing invitations to women known to have intellectual disability, most practices said they would send their usual standard letter and/or information leaflet.” (68) ^(p.89)^ This was reported to be “simpler for the practice, and avoided potentially insulting people by sending simplified letters or information leaflets to selected recipients.” (68) ^(p.89)^ This approach meant that patients required a family member to read the letter or that letters were discarded without being read (55, 68).

## 4 Discussion

This systematic review and meta-analysis provide compelling evidence of the extremely low prevalence of cervical screening among people with intellectual disability, and the significantly lower likelihood of this population receiving screening than people without intellectual disability. The review also highlighted the numerous and often unique barriers that hinder participation among this population.

The screening prevalence reported in our review, with just over one-third of people with intellectual disability having had a cervical screening test, was comparatively lower than has been reported in broader disability research (i.e., including people with other forms of disability) (25), although similar to results from a recent Swedish national database study of women with intellectual disability (24). Most studies have grouped people with intellectual disability together with people with psychosocial, sensory, physical, and other disabilities, which highlights the need for intellectual disability-focused research to adequately understand cervical screening participation among this population (70). Our review also highlighted that, with the exception of the Swedish study (24), studies linking national cancer screening registries and population demographics which can shine a spotlight on cervical screening participation at a population level for people with intellectual disability, were lacking. However, the WHO Global Strategy to Eliminate Cervical Cancer (5) has raised awareness of cervical screening data gaps and may lead to improvements, including in Australia where a government data linkage initiative, PLIDA (Person Level Integrated Data Asset) (71), will support the identification of people with disability within the National Cancer Screening Register.

Our review identified numerous factors that impacted participation in cervical screening by people with intellectual disability. Applying the socio-ecological model (SEM) to our analysis revealed multilevel factors influencing screening and enabled us to identify evidence gaps in the current literature (41). While most studies focused on individual and interpersonal level factors, there was less research examining organisational, community, and policy level influences. The majority of studies also looked at uptake and barriers to Pap test-based screening rather than HPV screening which was only introduced in 2017 in Australia (one of the earliest countries to switch from Pap tests to HPV testing) and, as expected, none included the relatively recent option of a self-collected sample for HPV testing without a speculum examination.

At the individual level, people with intellectual disability were reported to experience fear and anxiety about cervical screening, including fear of the test results, similar to previous research with women with other disabilities (72) and women without disability (73). However, for people with intellectual disability, fear and anxiety may be exacerbated by additional issues. These include a lack of accessible information and education to prepare for screening, and previous negative screening experiences due to poor healthcare provider communicative practices or having experienced a painful procedure where the healthcare provider failed to stop when requested to do so. Perceptions by healthcare providers, family members and support people that people with intellectual disability would not tolerate the cervical screening procedure can also preclude this population (19). The recent implementation in Australia of universal self-collection of an HPV test (whereby people can choose to take the sample themselves, or by a clinician if preferred, from the vagina without the need for a speculum examination) (74) offers promise to overcome some of these barriers posed by fears and anxiety. Raising awareness of this option, including amongst families, support people and disability organisations is essential (74).

Similarly, it was often assumed that people with intellectual disability would lack capacity to consent to cervical screening and healthcare providers frequently described the processes as difficult. Decisions to deny screening were made for people on the basis that they would not be able to consent, reflecting the findings of other intellectual disability health research (75). The shift in focus away from determining whether a person has the capacity to consent to medical procedures, to ensure that they are supported to make an informed decision through the use of accessible information and appropriate supports will hopefully lead to improvements in access to screening (75, 76). Training new generations of healthcare providers would also go some way in addressing the fear and anxiety experienced by women with intellectual disability through improved patient-provider communication and enhanced trust (77, 78).

Negative cultural discourse about people with intellectual disability contributed to misassumptions about cervical screening eligibility. This may include assumptions by healthcare providers and support people that people with intellectual disability are not engaging in consensual sexual relationships and a lack of recognition that this group is at significantly greater risk of sexual assault than the general community (22). Healthcare providers therefore may fail to ask relevant questions during a consultation, and may also focus on the person’s disability rather than broader healthcare needs (79). Training has been demonstrated to be an effective method to improve knowledge, broaden perspectives, and increase the confidence of healthcare providers in providing healthcare to people with intellectual disability (80). However, broader organisational and policy-level changes are also required to facilitate long-term change (15). For instance, strategies such as tailored communication about cervical screening, increased clinical appointment times and the availability of accessible information about cervical screening for people with intellectual disability (77) are essential to facilitate improved care.

Our review highlighted that support people could positively influence cervical screening participation for people with intellectual disability. Previous research has reported that support people do not always allow people with intellectual disability to speak for themselves in medical consultations, potentially preventing people with intellectual disability from exerting control over their healthcare needs (77). Healthcare providers’ over reliance on support people’s accounts when determining healthcare needs (81) is problematic, particularly when considering the pervasive misassumptions reported throughout the included literature that people with intellectual disability are sexually inactive. Denial of the sexuality of people with intellectual disability has far-reaching negative consequences including exclusion from sexual healthcare and education (22, 82). These findings underscore the need for cervical screening information and education that is inclusive of support people, and that emphasises the appropriate roles, to maximise positive screening outcomes.

People with intellectual disability may also not believe they are eligible for screening (46) due to a lack of visibility in health promotion information. In addition to the lack of accessible information about screening, systems that generate invitations to have a cervical screening test at a service or programmatic level also neglect to meet the needs people with intellectual disability.

Our review found that healthcare providers lacked information about the screening status of their clients, posing a significant challenge in ensuring participation in cervical screening. However, in Australia, a recent national digital cancer screening registry allows healthcare providers to access patients’ screening history (83), offering a promising solution to overcome this barrier. Enhanced coordination of preventative healthcare activities aims to improve equitable healthcare access and tailored support for people with intellectual disability. These findings emphasise the urgency for intellectual disability-focused initiatives to enhance participation in cervical screening programs.

There were a number of limitations in the published literature. Only a quarter of the studies included in this review focused specifically on cervical screening. Most studies included cervical screening within a broader focus on healthcare, which may fail to identify barriers that are particular to cervical screening. We found that less than a quarter of studies in this review sought data directly from people with intellectual disability. Most collected information through medical records, while qualitative studies often engaged with service providers such as disability support workers and healthcare providers. There is, therefore, a need to hear directly from people with intellectual disability to understand lived experiences of cervical screening (78). Furthermore, cervical cancer can impact anyone with a cervix (84), however studies focused primarily on white cisgender women. There is a need for future research to include all people with intellectual disability who have a cervix, including non-binary and gender diverse people who were assigned female at birth and trans men with a cervix (84) as well as Indigenous and Black people and people of colour.

### 4.1 Study limitations

There are several limitations to the present study. Only articles published in the English language were included in this review, representing high-income countries, and we did not explore grey literature. Although we used a comprehensive search strategy, terminology about intellectual disability differs internationally (85) and may pose limitations on the generalisability and comparability of our findings. We also did not assess the timeframe of cervical screening participation by subgroup or meta-regression, which could be a source of heterogeneity between the included studies.

### 4.2 Implications and future directions

Addressing disparity in cervical screening participation is essential to achieving the elimination of cervical cancer as a public health problem equitably and to improving the overall health and wellbeing of people with intellectual disability (5). Our findings highlight the need for multilevel strategies (41) to challenge negative cultural discourse about people with intellectual disability, which leads to stigmatisation and discrimination, and facilitates the continuation of systemic barriers. Inclusive, accessible, and supportive environments that encourage regular cervical screening with this population are needed. Furthermore, healthcare facilities should be designed and staffed to be inclusive of individuals with intellectual disability, ensuring that the physical, social, sensory and communication needs of people with intellectual disability are addressed (86). Collaboration between healthcare providers, disability service organisations, and policymakers is essential to implement targeted interventions and policies to improve cervical screening (86). Additionally, cervical screening information and public health campaigns should be tailored to reach people with intellectual disability, emphasising the importance of cervical screening. These resources and campaigns should utilise accessible formats, such as easy-to-read materials, visual aids, and multimedia resources, to ensure the information is accessible and engaging for this population (86). These efforts must be co-designed and community-led to ensure that the voices of people with intellectual disability with a diverse range of experiences are foregrounded in all initiatives aimed at enhancing cervical screening participation.

## 5 Conclusions

People with intellectual disability face significant disparities in cervical screening. Future research, policy, and practice initiatives are needed to address the barriers impacting cervical screening participation, facilitating long-term systemic change and social transformation. These efforts must be co-designed and community-led to effectively address the unique challenges faced by this population. By prioritising inclusive approaches, we can work towards achieving equity in both global and local endeavours aimed at eliminating cervical cancer.

## Author contributions

RP: Data curation, Formal analysis, Investigation, Methodology, Project administration, Writing – original draft, Writing – review & editing. MD: Conceptualization, Formal analysis, Funding acquisition, Investigation, Methodology, Supervision, Writing – original draft, Writing – review & editing. IS: Conceptualization, Formal analysis, Funding acquisition, Methodology, Supervision, Writing – review & editing. LT: Data curation, Methodology, Writing – review & editing, Writing – original draft. CB: Data curation, Methodology, Writing – review & editing. JL: Funding acquisition, Methodology, Writing – review & editing. HJ: Methodology, Writing – review & editing. EK: Methodology, Writing – review & editing. JU: Conceptualization, Funding acquisition, Methodology, Supervision, Writing – review & editing. SS: Funding acquisition, Methodology, Writing – review & editing. EC: Funding acquisition, Methodology, Writing – review & editing. AC: Funding acquisition, Methodology, Writing – review & editing. DB: Conceptualization, Funding acquisition, Methodology, Supervision, Writing – review & editing.

## Supplementary material

Supplementary tables and figures Easy Read Version - Cervical screening for people with intellectual disability
