# Supplementary material for: Cervical screening participation and access facilitators and barriers for people with intellectual disability: a systematic review and meta-analysis
Source: Front Psychiatry. 2024 Jul 26;15:1379497. doi: 10.3389/fpsyt.2024.1379497 (PMC11310793; doi:10.3389/fpsyt.2024.1379497)
Supplement: Supplementary tables and figures — Easy Read Version - Cervical screening for people with intellectual disability [file DataSheet_1.zip › Easy Read Version - Cervical screening for people with intellectual disability.DOCX]

**Easy Read Version**

**Cervical screening for people with intellectual disability**

| 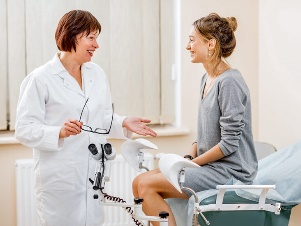 | This booklet is about people with intellectual disability and cervical screening  It talks about   - how many people did cervical screening - what helps - what does not help |
| --- | --- |
| **What we know** |  |
| 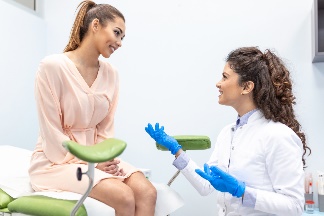  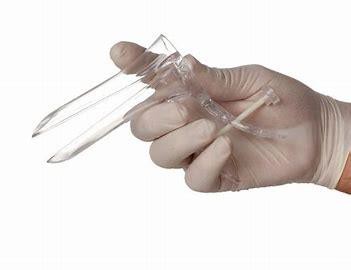 | Cervical screening is a test   - to check your **cervix** is healthy - that helps to stop **cervical cancer**   It used to be called   - a Pap test or a Pap smear |
| 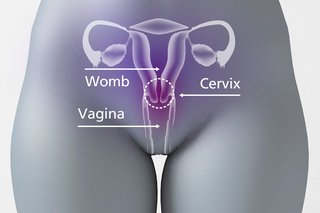 | The **cervix** is inside your body   - at the top of your **vagina** |
| 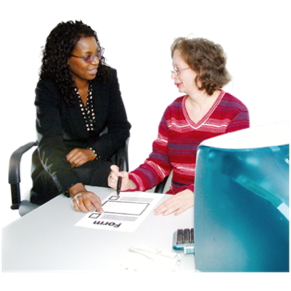 | Cervical cancer is   - one of the most stoppable cancers   caused **by** the **human papillomavirus infection**, **HPV for short** |
| 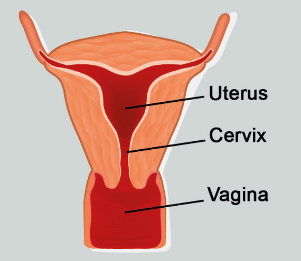 | **HPV** can cause changes in the cervix   - that could lead to cancer |
| 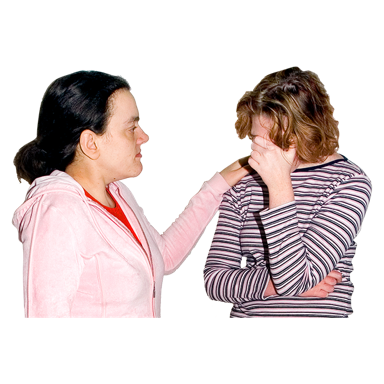 | Many people with intellectual disability   - have bad experiences with health care - have no choice about doing cervical screening |
| 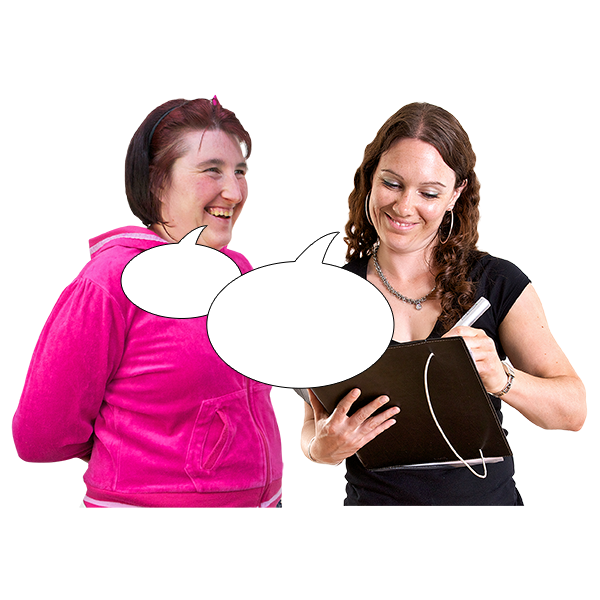 | We need to find out   - how many people with intellectual disability did cervical screening - what helps - what does not help |
| 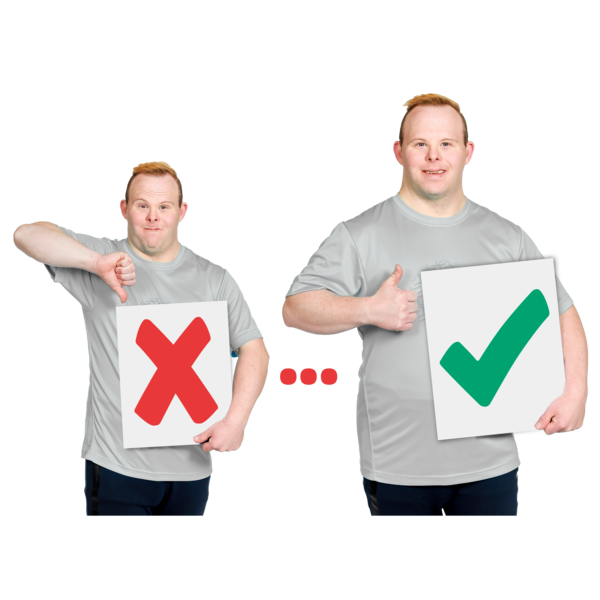 | This way, we can make cervical screening better   - for people with intellectual disability |

| **What we did** |  |
| --- | --- |
| **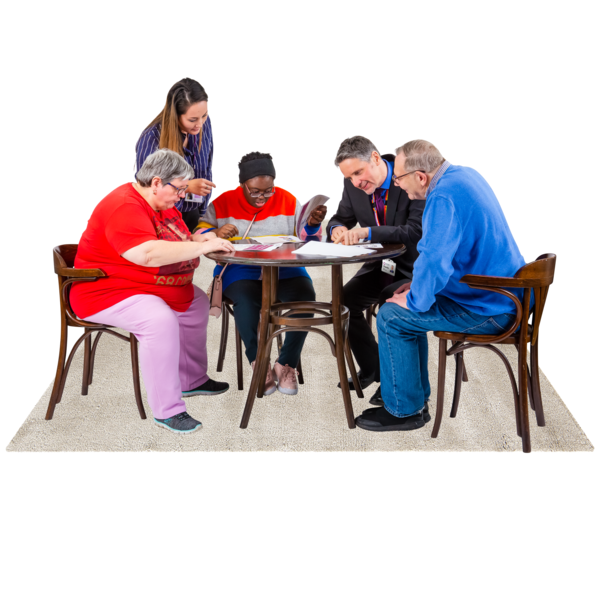** | We looked at what researchers already know about   - cervical screening for people with intellectual disability |
| 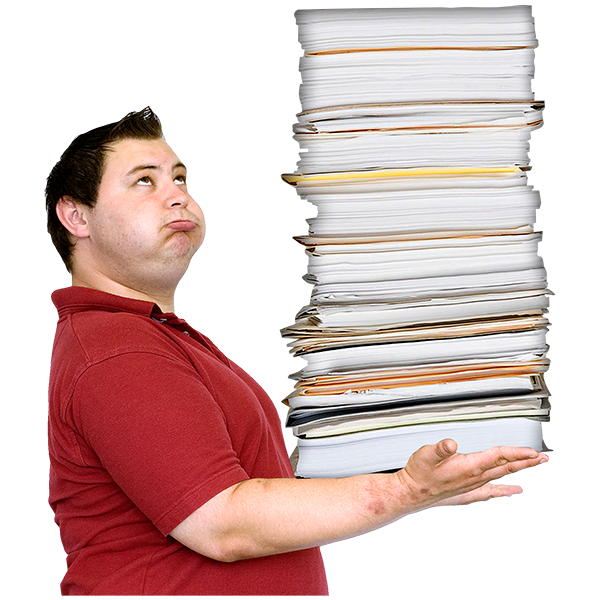 | We looked at 63 research studies  There were 42 studies about   - how many people did cervical screening |
| 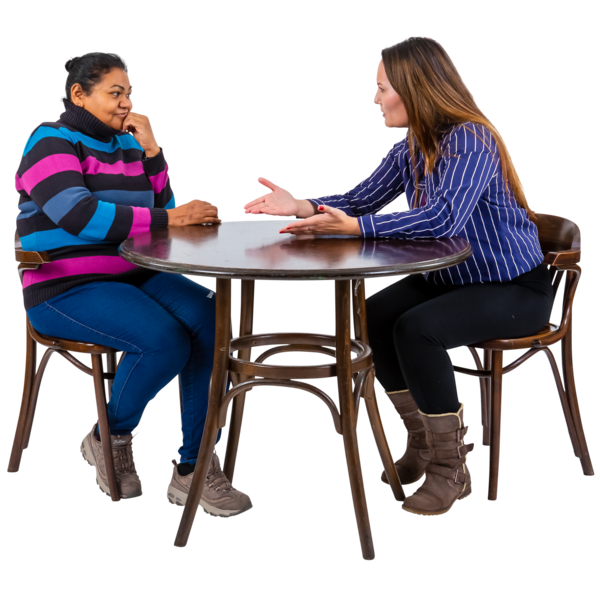 | 24 studies were about   - what helped people to have the test - what did not help |

| **What we found out** | |
| --- | --- |
| **How many people with intellectual disability did cervical screening?** | |
| **35%** | 35% of people with intellectual disability   - did cervical screening |
| 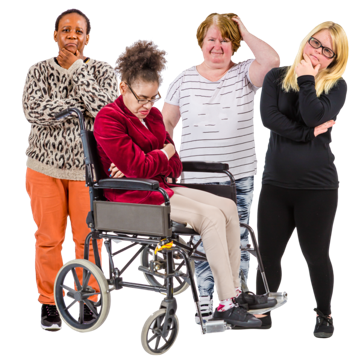 | People with intellectual disability are   - less likely to do cervical screening   than people without intellectual disability |
| **What helps or does not help people to do cervical screening?** | |
| **Idea 1 is about having a cervical screening test** | |
| 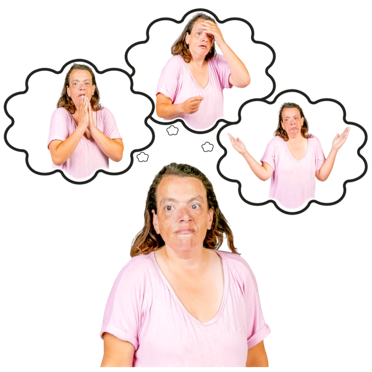 | People are worried the test will   - be painful - not be comfortable - be scary - remind them of sexual abuse - show they have cancer |
| 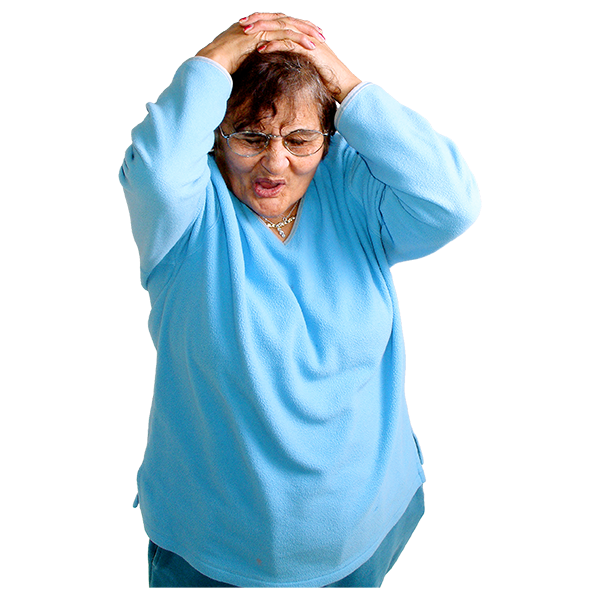 | Many people have bad experiences with health care |
| 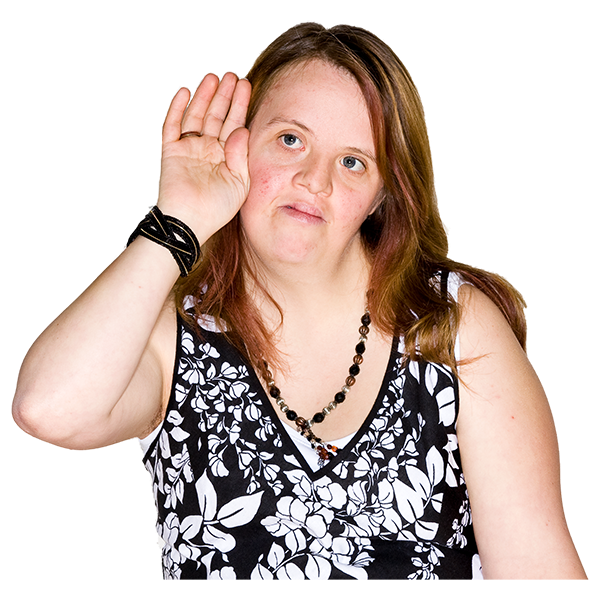 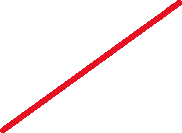 | They are   - not listened to - not treated fairly - told they do not need cervical screening - not given choices, instead doctors make decisions for them |
| 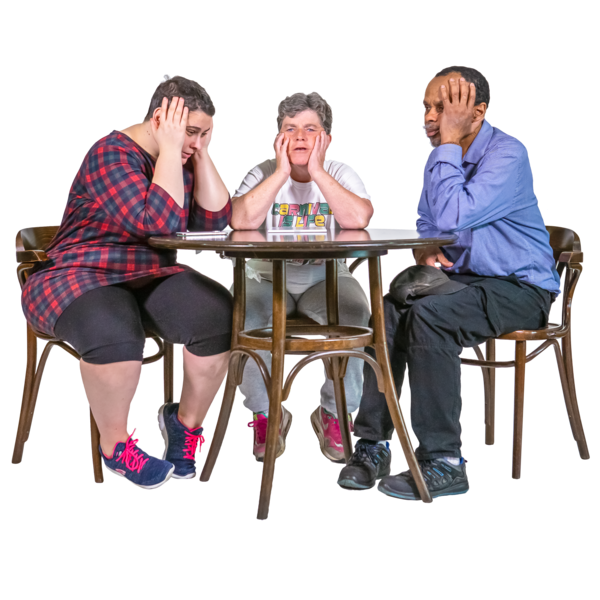 | This makes it very hard for people   - to feel good about cervical screening |
| 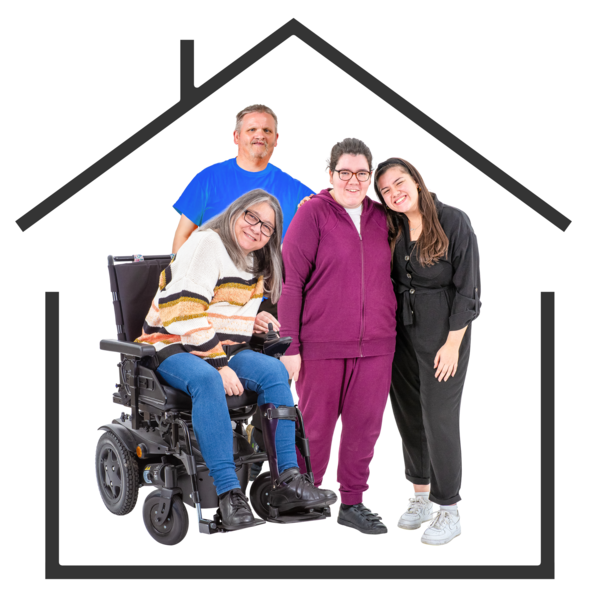 | People living with their parents were   - less likely to have a cervical screening test - than other people with intellectual disability |
| 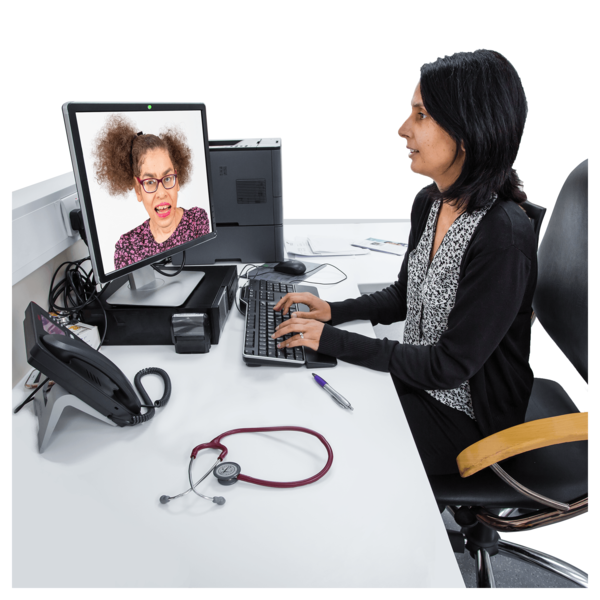 | People said they would have the test if they   - can talk openly to the doctor - trust the doctor - had a female doctor to do the test |

| **Idea 2 is about support people and health care workers** | |
| --- | --- |
| 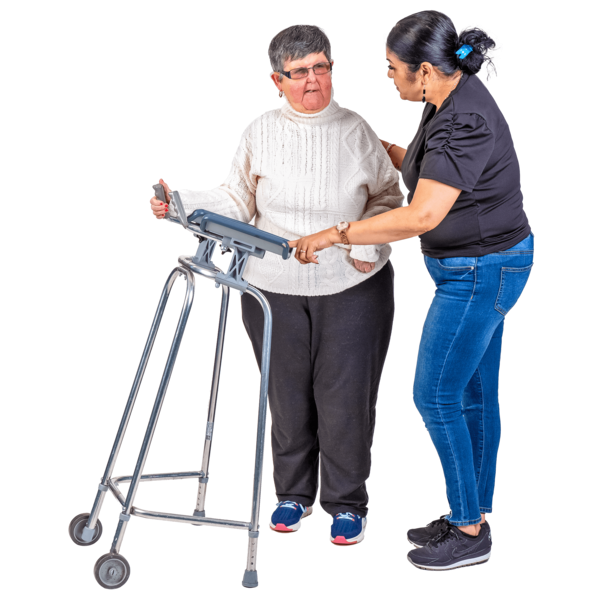 | Some of them think that people with intellectual disability   - do not need cervical screening |
| 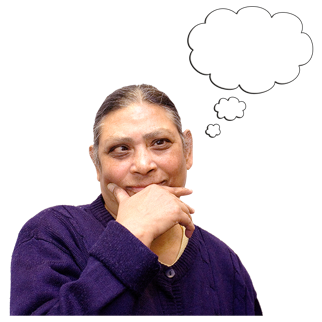 | They make a mistake to think that people   - never had sex - cannot understand about cervical screening - cannot lie still during cervical screening - cannot have screening done without medication |
| 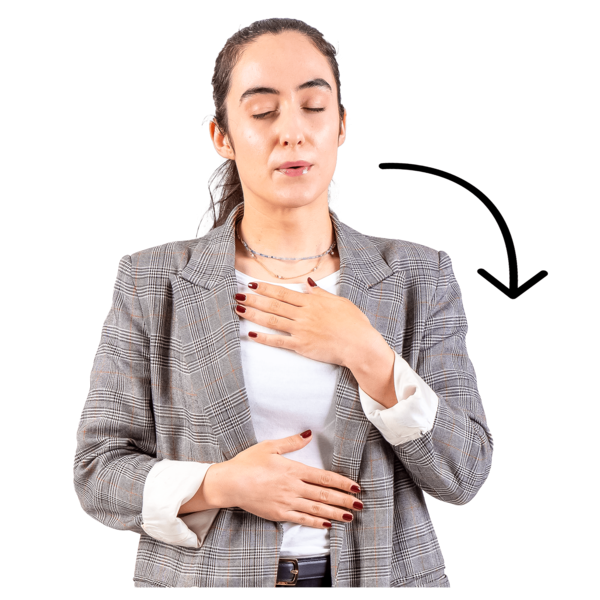 | During the test, support people can   - calm the person - hold the person’s hand - tell the person to breathe |
| 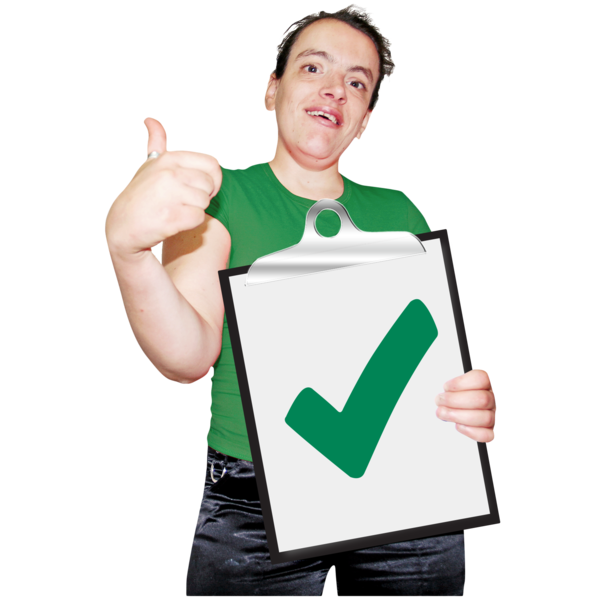 | It is important that the person   - feels good with the support person |
| 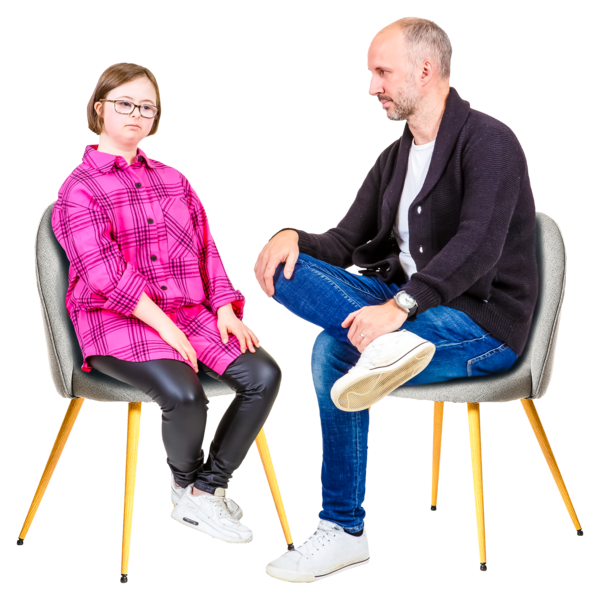 | Some support people   - get in the way - make cervical screening harder |
| 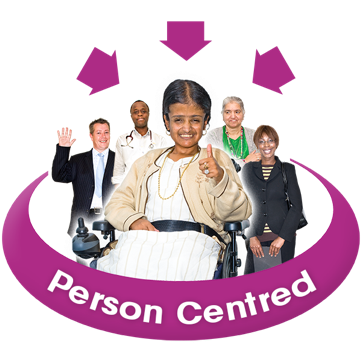 | Some health care workers   - get to know the person first - use pictures to explain cervical screening - show tools that will be used - make sure the person feels good and safe |
| 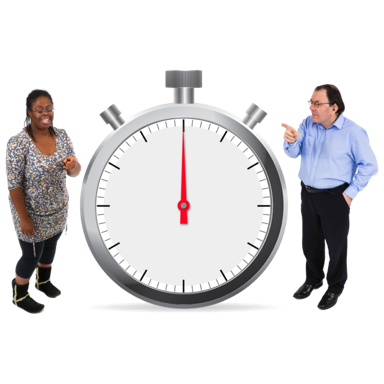  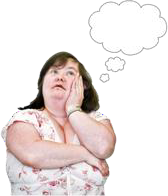 | There is not always enough time   - to do this in one appointment   People sometimes need more   - time to think - appointments   to make decisions about cervical screening |
| 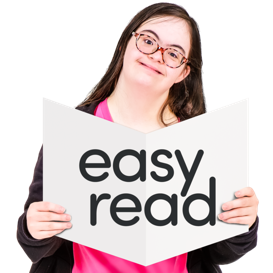 | Many health care workers do not   - know how to support people - give information that people can understand - give people choices |
| 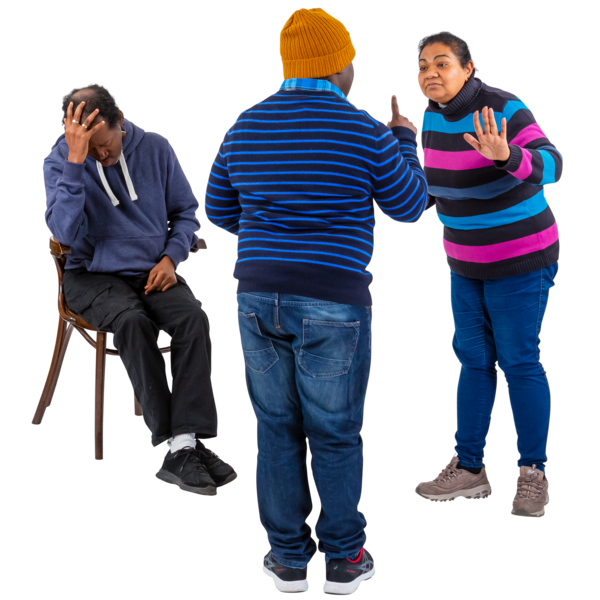 | Some health care workers talk to   - a support person - not to the person with intellectual disability |
| 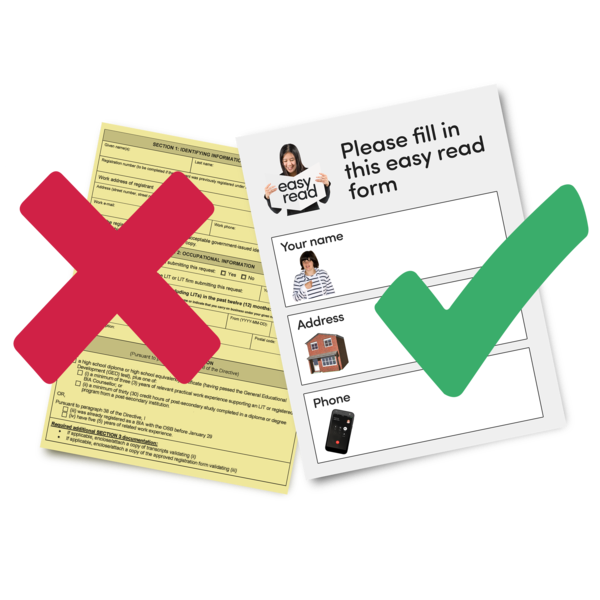 | Support people and health care workers need to   - explain things in plain English - help people make their own decisions |

| **Idea 3 is about disability and health care services** | |
| --- | --- |
| 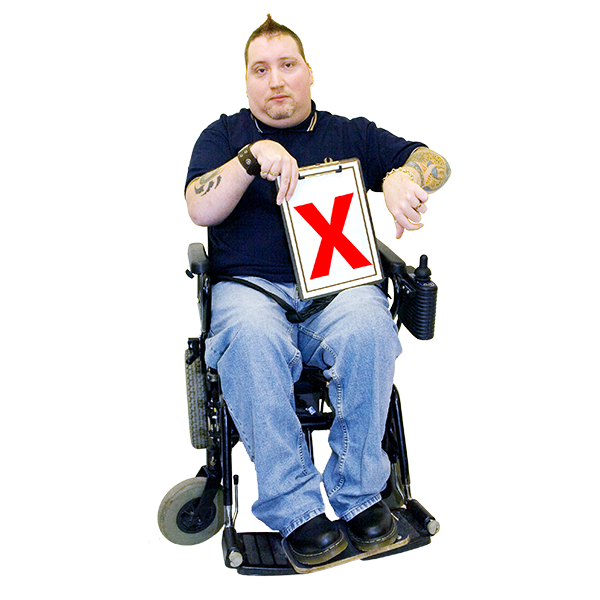 | Many health care services do not think about   - needs of people with intellectual disability |
| 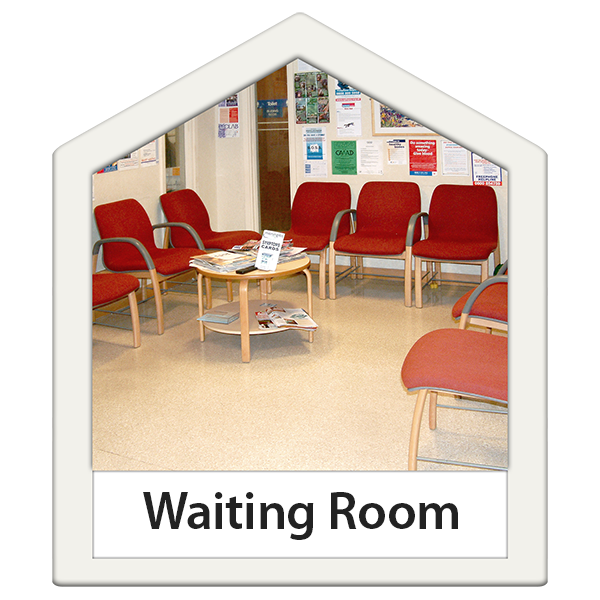 | Health care services need to have   - quiet waiting rooms - long appointment times - appointment times that suit the person |
| 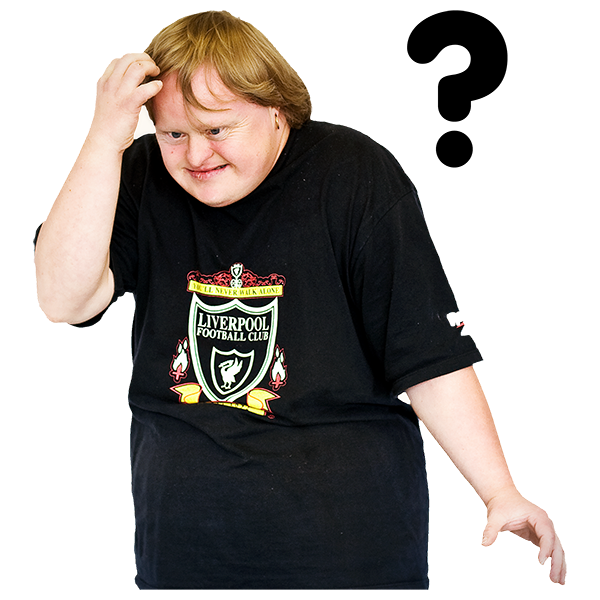 | Some disability services do not know   - if the person needs cervical screening |
| 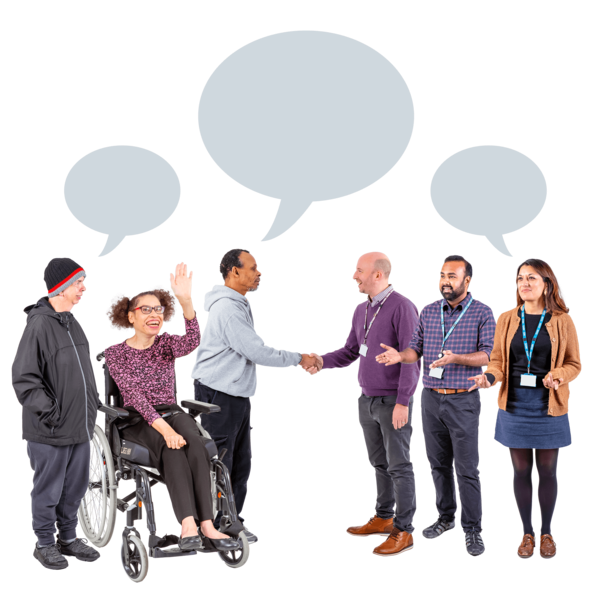 | Disability and health services   - need to work together   so the needs of the person are met |
|  |  |

| **Idea 4 is about problems in the health system** | |
| --- | --- |
| 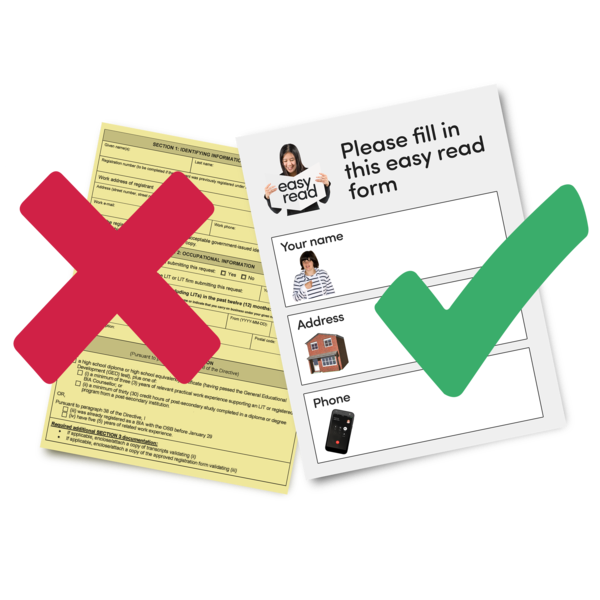 | Information about cervical screening   - is often hard to understand |
| 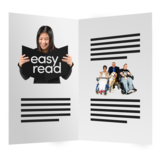 | People with intellectual disability are not shown   - in booklets about cervical screening |
| 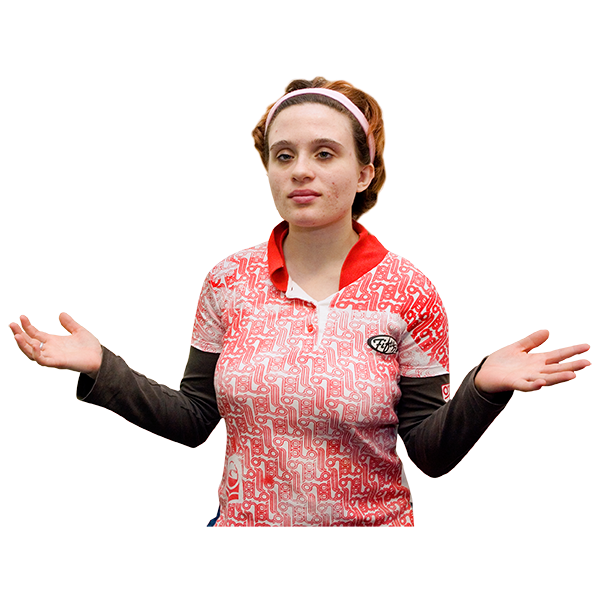 | This makes it very hard for people to know   - that they need cervical screening - how and where to get it |
| **How we can make things better** | |
| 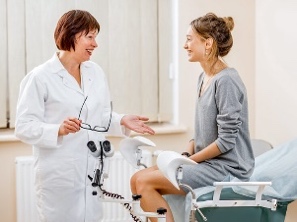 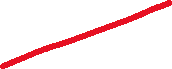 | Most people with intellectual disability   - did not have cervical screening |
| 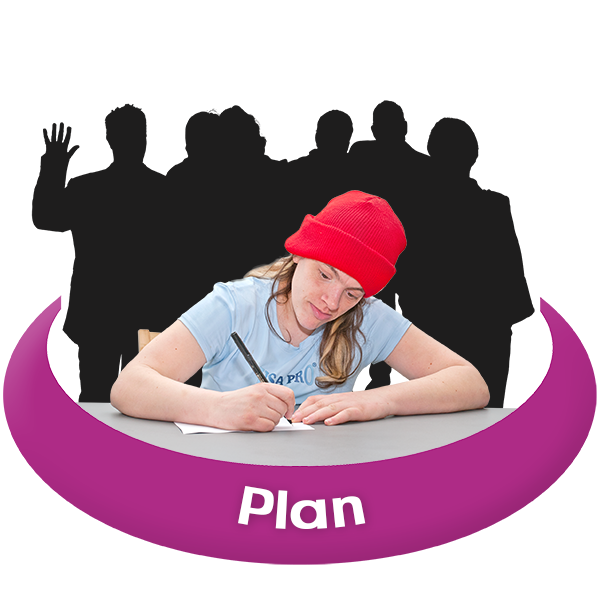 | People with intellectual disability need to be part of   - cervical screening plans |
| 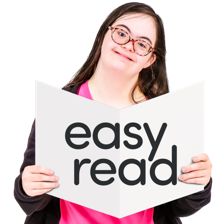 | People need information that they can understand   - to make their own decision about cervical screening |
| 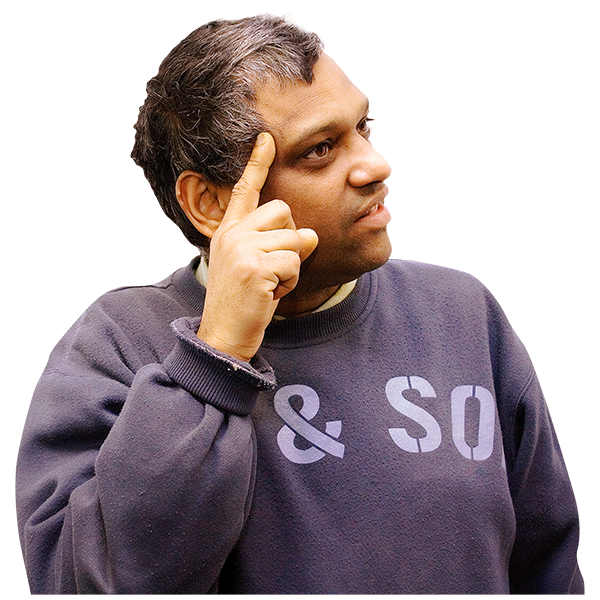 | Support people and health care workers need to know   - that people with intellectual disability have sex |
| 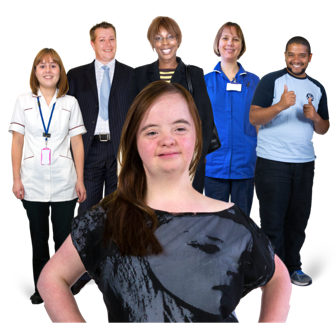 | They also need to know that people with intellectual disability   - have bad experiences with health care - can learn about cervical screening - can make a decision to have cervical screening - can have cervical screening |
| 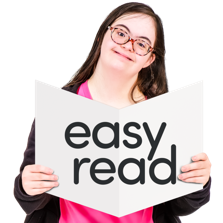  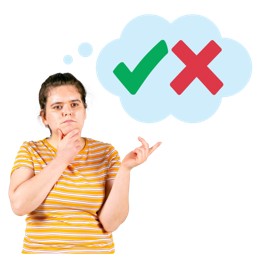 | We must teach support people and health care workers to   - use Easy Read information - give choices to people - explain things so people can understand - work together to best support people |
| 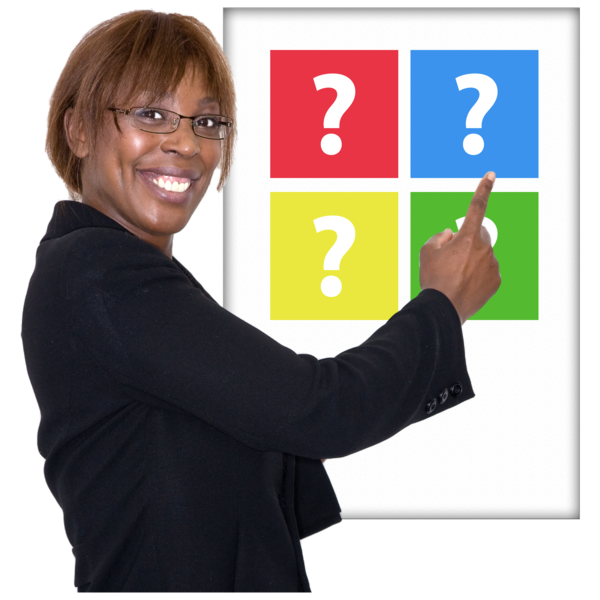 | This will help people with intellectual disability   - make choices about cervical screening |
| 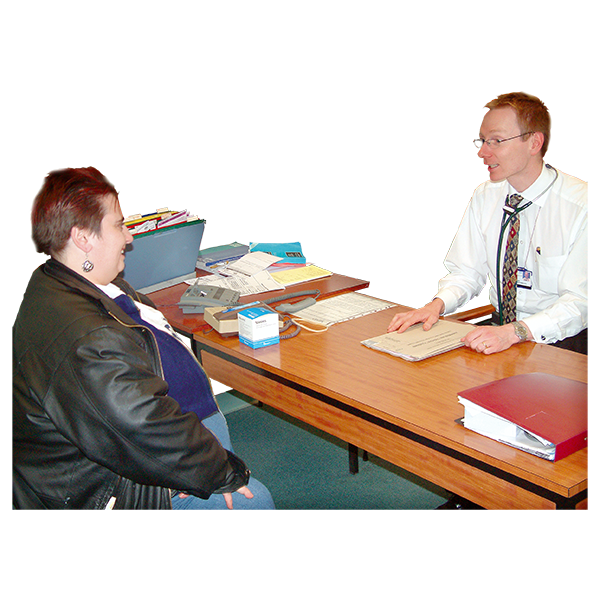 | Health care workers need to   - speak to the person   not only the support people |
| 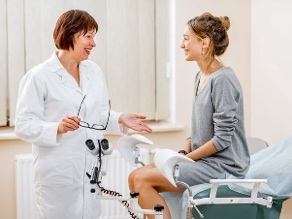 | Support people need to know if the person   - had cervical screening |
| 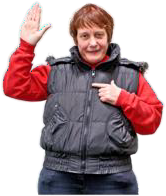  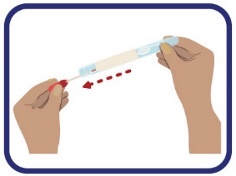 | Health care workers need to give people   - a choice about cervical screening   There is now a choice for people   - to do the test themselves   This is called self-collection |
| 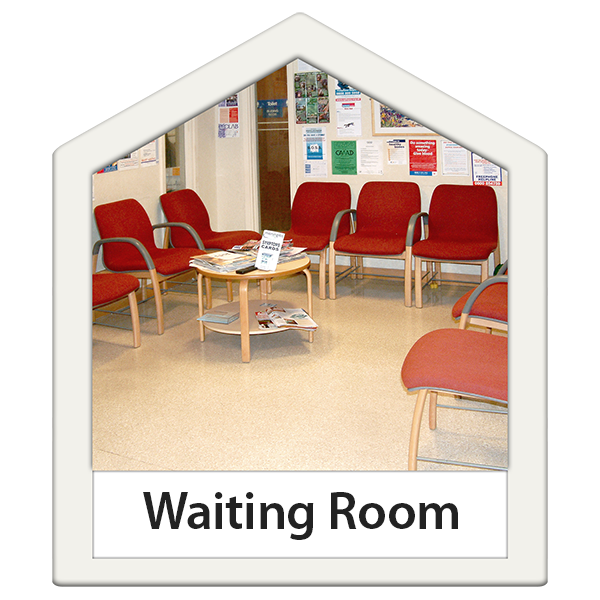 | Health care services need to   - make sure waiting rooms are friendly - allow more time - train health care workers |
| 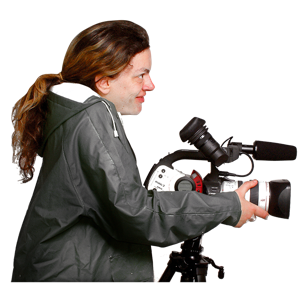 | The health system needs to give information   - about cervical screening in ways people can understand   This can be Easy Read or videos that include   - people with intellectual disability in talking about cervical screening to others |
| 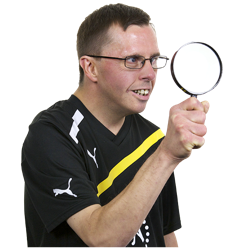 | We need to find out more about   - what people think about cervical screening - how people make choices about cervical screening |
| 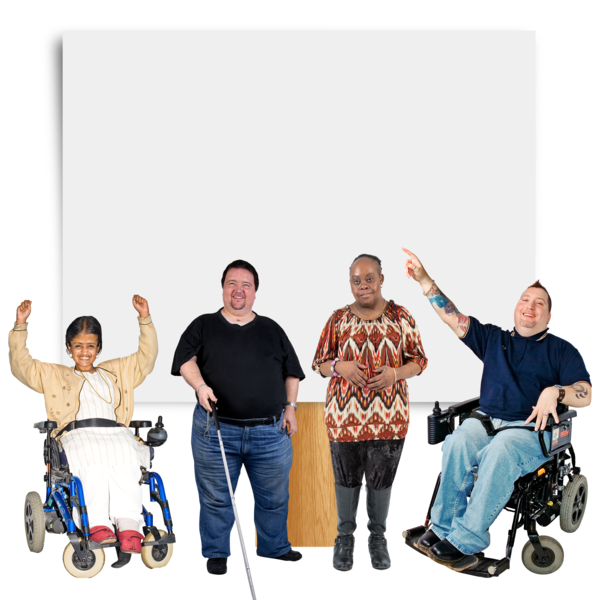 | All people with a cervix   - have a right to be included in research |
| 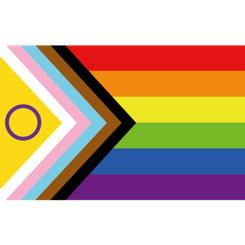  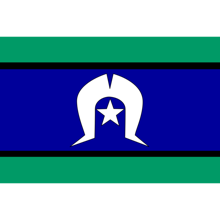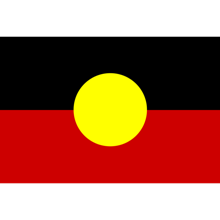 | This includes   - trans men - non-binary and gender diverse people - Indigenous and Black people and people of colour |
| 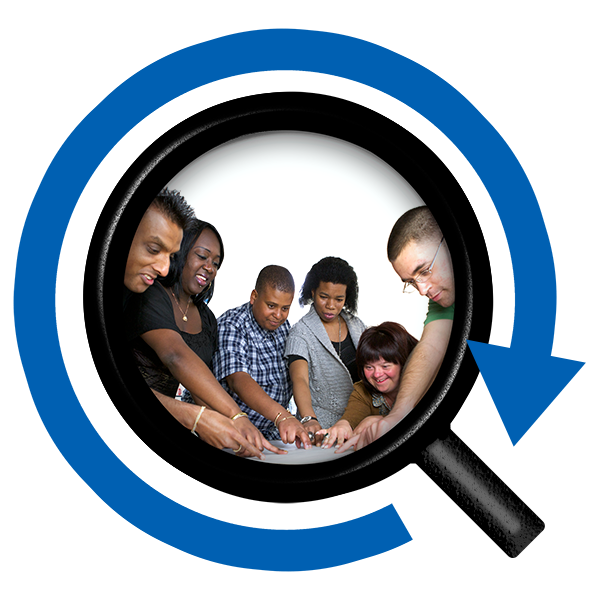 | This should be **inclusive research** |
| 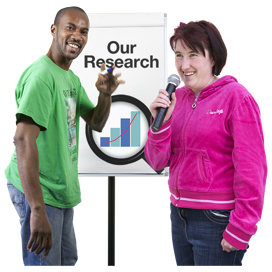 | **Inclusive research** is when researchers   - work together with people with intellectual disability   to find out more |
| 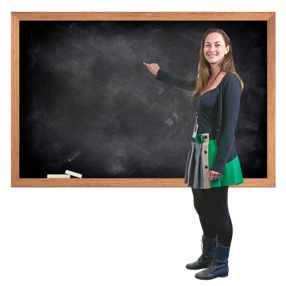 | We can teach support people and health care workers to   - support people better - help people make their own choices about cervical screening |

| **More information** |  |
| --- | --- |
| 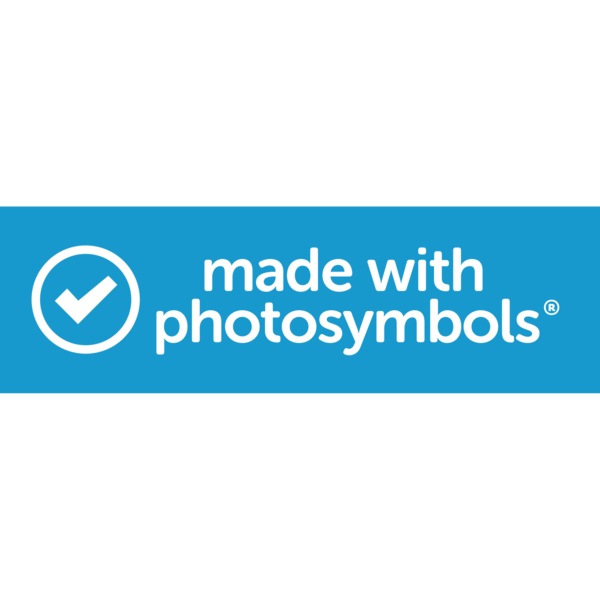 | The ScreenEQUAL team made this booklet  It was made with Photosymbols |
| 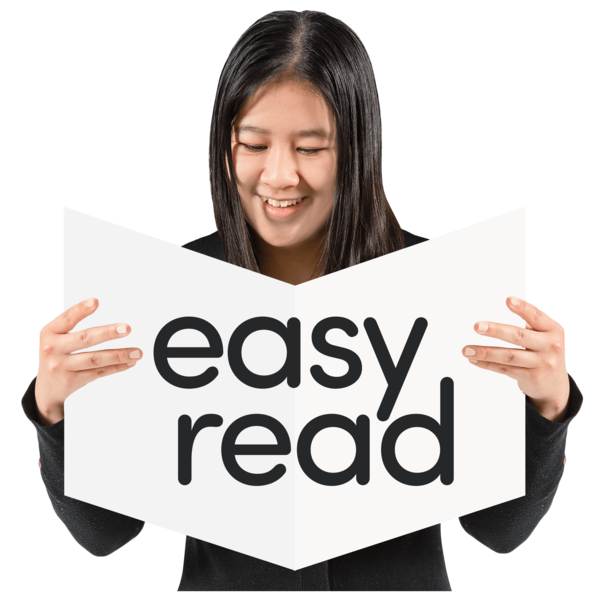 | This booklet is an Easy Read summary about   - some of our research |
| 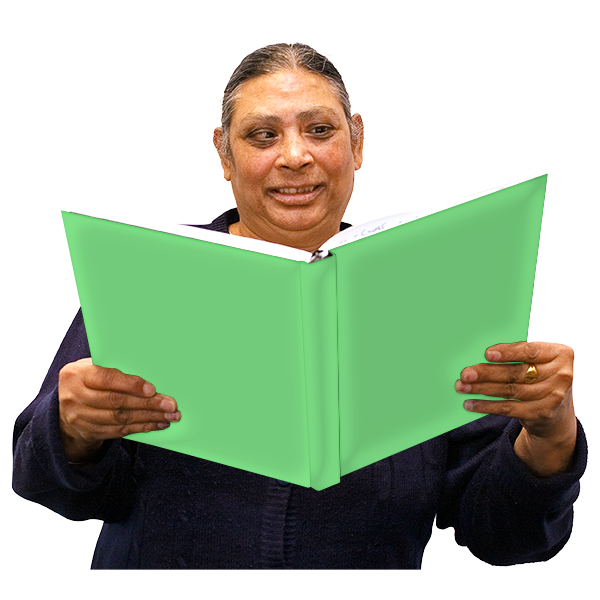 | The full article is called:  Cervical screening participation and access facilitators and barriers for people with intellectual disability: A systematic review and meta-analysis. |
| 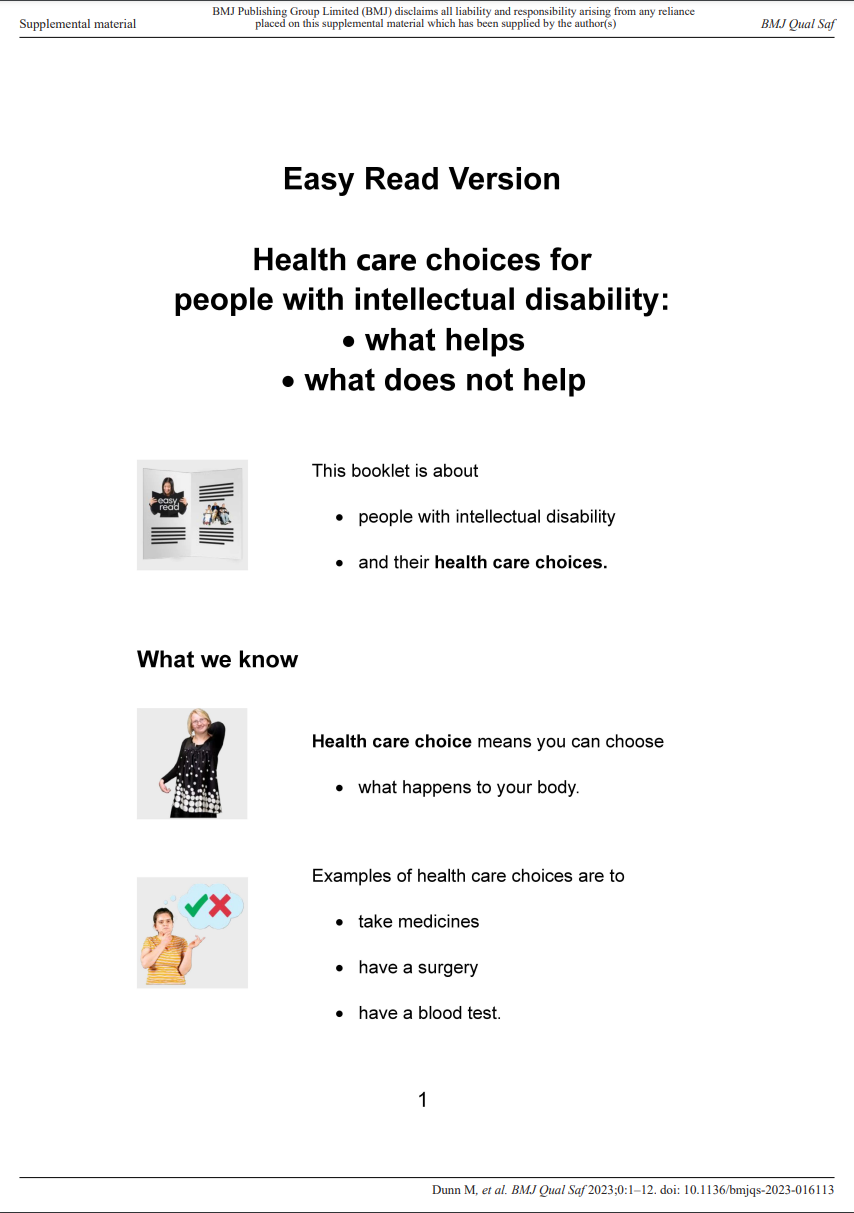 | To make this booklet we looked at other Easy Read research  We looked at   - Health care choices for people with intellectual disability   Go to  <https://doi.org/10.1136/bmjqs-2023-016113> |

**Suggested citation:**

Power, R., Loblinzk, J., Strnadová, I., David, M., Touyz, L., Baskin, C., Jolly, H., Kennedy, H., Ussher, J., Sweeney, S., Chang, E., Carter, A., Bateson, D. (under review) Cervical screening for people with intellectual disability. Easy Read version. Frontiers In Psychiatry. <<publication details pending>>
