# Supplementary material for: Cervical screening participation and access facilitators and barriers for people with intellectual disability: a systematic review and meta-analysis
Source: Front Psychiatry. 2024 Jul 26;15:1379497. doi: 10.3389/fpsyt.2024.1379497 (PMC11310793; doi:10.3389/fpsyt.2024.1379497)
Supplement: Supplementary tables and figures — Easy Read Version - Cervical screening for people with intellectual disability [file DataSheet_1.zip › Supplementary tables and figures.docx]

***Supplementary Material***

Cervical screening participation and access facilitators and barriers for people with intellectual disability: A systematic review and meta-analysis.

**Rosalie Power^1^, Michael David^2,3^ Lauren Touyz^2^, Caroline Baskin^4^, Julie Loblinzk OAM^4^, Iva** **Strnadová^4^, Heather Jolly^5^, Elizabeth Kennedy^1^, Jane Ussher^1^, Sally Sweeney^5^, Ee-Lin Chang^5^, Allison Carter^6^, Deborah Bateson^2*^**

*** Correspondence:** Professor Deborah Bateson, deborah.bateson@sydney.edu.au

# Supplementary Figures

## Supplementary Figure 1. Forest plot for screening prevalence for people with intellectual disability by the Year of publication.

## Supplementary Figure 2. Forest plot for screening prevalence for people with intellectual disability by Continent.

## Supplementary Figure 3: Forest plot of the association between screening participation and intellectual disability among women by Year of Publication

## Supplementary Figure 4: Forest plot by Continent for the association between screening participation for people with intellectual disability compared to people without intellectual disability

# Supplementary Tables

## Supplementary Table 1. Search strategy.

| **Focus** | **Title-Abs search** |
| --- | --- |
| Cervical screening | AND (TITLE-ABS ( ( ( pap OR Papanicolaou ) W/2 ( smear OR test ) ) OR ( ( cancer* OR neoplas* OR smear ) W/2 cervi* ) OR ( ( "cervi* cancer*" OR "cervi* neoplas*" ) W/2 ( screen* OR test OR detect* ) ) OR "human papillomavirus" OR "human papilloma virus" ) ) |
| Population | AND (TITLE-ABS ( ( ( intellectual* OR learning OR developmental* OR cognitive* ) W/2 ( disab* OR disord* OR impair* OR delay* ) ) OR "Down* syndrome*" OR "mental* retard*" ) ) |

## Supplementary Table 2. Standard Quality Assessment Scores

| **Study reference** | **Score** |
| --- | --- |
| Agaronnik, Pendo ^1^ | 0.85 |
| Armin, Williamson ^2^ | 0.85 |
| Bershadsky, Taub ^3^ | 0.96 |
| Biswas, Whalley ^4^ | 0.94 |
| Breau, Thorne ^5^ | 0.10 |
| Breau, Thorne ^6^ | 0.80 |
| Broughton and Thomson ^7^ | 0.10 |
| Brown, Plourde ^8^ | 0.10 |
| Brown, Jacobstein ^9^ | 0.10 |
| Bussiere, Le Vaillant ^10^ | 0.10 |
| Byrne, Ware ^11^ | 0.10 |
| Chen, Kung ^12^ | 0.10 |
| Cobigo, Ouellette-Kuntz ^13^ | 0.10 |
| Conder, Mirfin-Veitch ^14^ | 0.80 |
| Cooper, McConnachie ^15^ | 0.96 |
| Durbin, Selick ^16^ | 0.91 |
| Folch, Salvador-Carulla ^17^ | 0.90 |
| Fortney and Tasse ^18^ | 0.10 |
| Glover, Christie ^19^ | 0.80 |
| Haider, Ansari ^20^ | 0.10 |
| Havercamp, Scandlin ^21^ | 0.96 |
| Havercamp and Scott ^22^ | 0.10 |
| Hu, Wang ^23^ | 0.10 |
| Huang, Tsai ^24^ | 0.10 |
| Hughes-McCormack, Greenlaw ^25^ | 0.10 |
| Humphrey, Horn ^26^ | 0.10 |
| Kellen, Nuyens ^27^ | 0.94 |
| Kerr, Richards ^28^ | 0.10 |
| Langan, Whitfield ^29^ | 0.75 |
| Lennox, Bain ^30^ | 0.10 |
| Lin, Lin ^31^ | 0.91 |
| Lin, Sung ^32^ | 0.94 |
| Lin, Lin ^33^ | 0.95 |
| Lloyd and Coulson ^34^ | 0.80 |
| Maltais, Morin ^35^ | 0.10 |
| Marquis, Lunsky ^36^ | 0.10 |
| Olsen, Søndenaa ^37^ | 0.10 |
| Osborn, Horsfall ^38^ | 0.10 |
| Ouellette-Kuntz, Cobigo ^39^ | 0.10 |
| Palmer, Heung ^40^ | 0.10 |
| Parish, Saville ^41^ | 0.10 |
| Parish, Moss ^42^ | 0.85 |
| Parish, Rose ^43^ | 0.10 |
| Parish, Swaine ^44^ | 0.10 |
| Parish, Swaine ^45^ | 0.10 |
| Plourde, Brown ^46^ | 0.10 |
| Rees ^47^ | 0.89 |
| Reichard, Stolzle ^48^ | 0.10 |
| Reichard and Fox ^49^ | 0.10 |
| Reynolds, Stanistreet ^50^ | 0.91 |
| Shin, Lee ^51^ | 0.10 |
| Smith, Applebaum ^52^ | 0.95 |
| Son, Parish ^53^ | 0.10 |
| Steele, Townsend ^54^ | 0.10 |
| Stein and Allen ^55^ | 0.80 |
| Stein ^56^ | 0.83 |
| Swaine, Dababnah ^57^ | 0.80 |
| Swaine, Parish ^58^ | 0.10 |
| Sykes, McGeechan ^59^ | 0.89 |
| Wicks ^60^ | 0.10 |
| Wood and Douglas ^61^ | 0.10 |
| Xu, McDermott ^62^ | 0.10 |
| Yen, Kung ^63^ | 0.10 |

## Supplementary Table 3. Meta-regression for the association between the moderators of Publication Year and Continent, and screening prevalence

| **Moderator** | $\boldsymbol{\beta}$ | **95% CI** | **P-value** | **Overall**  **P-value** |
| --- | --- | --- | --- | --- |
| **Continent** |  |  |  | <0.01 |
| Asia | -2.21 | -3.15 - -1.28 | <0.01 |  |
| Europe | -0.90 | -1.62 - -0.17 | 0.02 |  |
| North America (Reference) |  |  |  |  |
| Oceania | -2.41 | -3.64 - -1.17 | <0.01 |  |
|  |  |  |  |  |
| **Year of Publication** |  |  |  | 0.31 |
| 1996 – 2002 (Reference) |  |  |  |  |
| 2003 - 2009 | 0.87 | -0.55 - 2.28 | 0.23 |  |
| 2010 - 2016 | 0.90 | -0.30 - 2.10 | 0.14 |  |
| 2017 - 2023 | 0.41 | -0.78 - 1.60 | 0.50 |  |

$\beta$: regression coefficient; CI: confidence interval

## Supplementary Table 4: Meta-regression for associations between Type of Analysis, Continent, Publication Year and screening participation for people with intellectual disability compared to people without intellectual disability

| **Moderator** | $\boldsymbol{\beta}$ | **95% CI** | **P-value** | **Overall**  **P-value** |
| --- | --- | --- | --- | --- |
| **Adjusted Analysis** |  |  |  | 0.45 |
| No (Reference) |  |  |  |  |
| Yes | 0.37 | -0.60 – 1.35 | 0.45 |  |
|  |  |  |  |  |
| **Continent** |  |  |  | 0.42 |
| Asia | -0.03 | -1.44 – 1.38 | 0.97 |  |
| Europe | -0.49 | -1.46 – 0.47 | 0.32 |  |
| North America (Reference) |  |  |  |  |
| Oceania | -1.49 | -3.37 – 0.40 | 0.12 |  |
|  |  |  |  |  |
| **Year of Publication** |  |  |  |  |
| 2004 - 2013 (Reference) |  |  |  |  |
| 2014 - 2023 | -0.02 | -0.96 – 0.92 | 0.97 | 0.97 |

$\beta$: regression coefficient; CI: confidence interval

# References

1. Agaronnik N, Pendo E, Lagu T, DeJong C, Perez-Caraballo A, Iezzoni LI. Ensuring the Reproductive Rights of Women with Intellectual Disability. J Intellect Dev Disabil. 2020;45(4):365-76.

2. Armin JS, Williamson HJ, Begay A, Etcitty J, Attakai A, Russell K, et al. Adapting a Cancer Screening Education Program for Native American Women with Disabilities. Int J Environ Res Public Health. 2022;19(15).

3. Bershadsky J, Taub S, Engler J, Moseley CR, Lakin KC, Stancliffe RJ, et al. Place of residence and preventive health care for intellectual and developmental disabilities services recipients in 20 states. Public Health Rep. 2012;127(5):475-85.

4. Biswas M, Whalley H, Foster J, Friedman E, Deacon R. Women with learning disability and uptake of screening: audit of screening uptake before and after one to one counselling. J Public Health (Oxf). 2005;27(4):344-7.

5. Breau G, Thorne S, Baumbusch J, Hislop TG, Kazanjian A. Primary Care Providers' Attitudes Towards Recommending Cancer Screening to Patients With Intellectual Disability: A Cross-Sectional Survey. Inclusion. 2020;8(3):185-93.

6. Breau G, Thorne S, Baumbusch J, Hislop TG, Kazanjian A. Family physicians' and trainees' experiences regarding cancer screening with patients with intellectual disability: An interpretive description study. J Intellect Disabil. 2023;27(1):250-65.

7. Broughton S, Thomson K. Women with learning disabilities: risk behaviours and experiences of the cervical smear test. J Adv Nurs. 2000;32(4):905-12.

8. Brown HK, Plourde N, Ouellette-Kuntz H, Vigod S, Cobigo V. Brief report: cervical cancer screening in women with intellectual and developmental disabilities who have had a pregnancy. J Intellect Disabil Res. 2016;60(1):22-7.

9. Brown M, Jacobstein D, Yoon IS, Anthony B, Bullock K. Systemwide Initiative Documents Robust Health Screening for Adults With Intellectual Disability. Intellect Dev Disabil. 2016;54(5):354-65.

10. Bussiere C, Le Vaillant M, Pelletier-Fleury N. Screening for cervical cancer: What are the determinants among adults with disabilities living in institutions? Findings from a National Survey in France. Health Policy. 2015;119(6):794-801.

11. Byrne JH, Ware RS, Lennox NG. Health actions prompted by health assessments for people with intellectual disability exceed actions recorded in general practitioners' records. Aust J Prim Health. 2015;21(3):317-20.

12. Chen CY, Kung PT, Chiu LT, Tsai WC. Comparison of Cervical Cancer Screening Used between Individuals with Disabilities and Individuals without Disabilities. Healthcare (Basel). 2023;11(10).

13. Cobigo V, Ouellette-Kuntz H, Balogh R, Leung F, Lin E, Lunsky Y. Are cervical and breast cancer screening programmes equitable? The case of women with intellectual and developmental disabilities. J Intellect Disabil Res. 2013;57(5):478-88.

14. Conder J, Mirfin-Veitch B, Payne D, Channon A, Richardson G. Increasing the participation of women with intellectual disabilities in women's health screening: a role for disability support services. Research and Practice in Intellectual and Developmental Disabilities. 2018;6(1):86-96.

15. Cooper SA, McConnachie A, Allan LM, Melville C, Smiley E, Morrison J. Neighbourhood deprivation, health inequalities and service access by adults with intellectual disabilities: a cross-sectional study. J Intellect Disabil Res. 2011;55(3):313-23.

16. Durbin J, Selick A, Casson I, Green L, Perry A, Chacra MA, et al. Improving the quality of primary care for adults with intellectual and developmental disabilities: Value of the periodic health examination. Can Fam Physician. 2019;65(Suppl 1):S66-S72.

17. Folch A, Salvador-Carulla L, Vicens P, Cortes MJ, Irazabal M, Munoz S, et al. Health indicators in intellectual developmental disorders: The key findings of the POMONA-ESP project. J Appl Res Intellect Disabil. 2019;32(1):23-34.

18. Fortney S, Tasse MJ. Urbanicity, Health, and Access to Services for People With Intellectual Disability and Developmental Disabilities. Am J Intellect Dev Disabil. 2021;126(6):492-504.

19. Glover G, Christie A, Hatton C. Access to cancer screening by people with learning disabilities in England 2012/13: information from the Joint Health and Social Care Assessment Framework. Tizard Learning Disability Review. 2014;19(4):194-8.

20. Haider SI, Ansari Z, Vaughan L, Matters H, Emerson E. Health and wellbeing of Victorian adults with intellectual disability compared to the general Victorian population. Res Dev Disabil. 2013;34(11):4034-42.

21. Havercamp SM, Scandlin D, Roth M. Health Disparities Among Adults with Developmental Disabilities, Adults with other Disabilities, and Adults Not Reporting Disability in North Carolina. Public Health Reports. 2004;119.

22. Havercamp SM, Scott HM. National health surveillance of adults with disabilities, adults with intellectual and developmental disabilities, and adults with no disabilities. Disabil Health J. 2015;8(2):165-72.

23. Hu K, Wang J, Sparen P, Herweijer E, Sjolander A, Adami HO, et al. Invasive cervical cancer, precancerous lesions, and cervical screening participation among women with mental illness in Sweden: a population-based observational study. Lancet Public Health. 2023;8(4):e266-e75.

24. Huang KH, Tsai WC, Kung PT. The use of Pap smear and its influencing factors among women with disabilities in Taiwan. Res Dev Disabil. 2012;33(2):307-14.

25. Hughes-McCormack L, Greenlaw N, McSkimming P, McCowan C, Ross K, Allan L, et al. Changes over time in the management of long-term conditions in primary health care for adults with intellectual disabilities, and the healthcare inequality gap. J Appl Res Intellect Disabil. 2021;34(2):634-47.

26. Humphrey KN, Horn PS, Olshavsky L, Reebals L, Standridge SM. Well-woman care and HPV vaccination rates in women with Rett syndrome. Disabil Health J. 2021;14(1):100982.

27. Kellen E, Nuyens C, Molleman C, Hoeck S. Uptake of cancer screening among adults with disabilities in Flanders (Belgium). J Med Screen. 2020;27(1):48-51.

28. Kerr MP, Richards D, Glover G. Primary Care for People with an Intellectual Disability - A Group Practice Survey. Journal of Applied Research in Intellectual Disabilities. 1996;9(4):347-52.

29. Langan J, Whitfield M, Russell O. Paid and unpaid carers: their role in and satisfaction with primary health care for people with learning disabilities. Health & Social Care in the Community. 1994;2(6):357-65.

30. Lennox N, Bain C, Rey-Conde T, Purdie D, Bush R, Pandeya N. Effects of a comprehensive health assessment programme for Australian adults with intellectual disability: a cluster randomized trial. Int J Epidemiol. 2007;36(1):139-46.

31. Lin LP, Lin JD, Sung CL, Liu TW, Liu YL, Chen LM, et al. Papanicolaou smear screening of women with intellectual disabilities: a cross-sectional survey in Taiwan. Res Dev Disabil. 2010;31(2):403-9.

32. Lin JD, Sung CL, Lin LP, Liu TW, Lin PY, Chen LM, et al. Perception and experience of primary care physicians on Pap smear screening for women with intellectual disabilities: a preliminary finding. Res Dev Disabil. 2010;31(2):440-5.

33. Lin LP, Lin JD, Chu CM, Chen LM. Caregiver attitudes to gynaecological health of women with intellectual disability. J Intellect Dev Disabil. 2011;36(3):149-55.

34. Lloyd JL, Coulson NS. The role of learning disability nurses in promoting cervical screening uptake in women with intellectual disabilities: A qualitative study. J Intellect Disabil. 2014;18(2):129-45.

35. Maltais J, Morin D, Tasse MJ. Healthcare services utilization among people with intellectual disability and comparison with the general population. J Appl Res Intellect Disabil. 2020;33(3):552-64.

36. Marquis S, Lunsky Y, McGrail KM, Baumbusch J. Population-level use of gynecological health services by female youth with intellectual/developmental disabilities in British Columbia Canada. Disability and Health Journal. 2023;16(3).

37. Olsen MI, Søndenaa E, Langballe EM, Halvorsen MB, Wilhelmsen P, Bautz-Holter E, et al. Use of health and dental care services in adults with intellectual disability in relation to age and intellectual disability levels. Journal of Intellectual & Developmental Disability. 2022;48(2):172-83.

38. Osborn DP, Horsfall L, Hassiotis A, Petersen I, Walters K, Nazareth I. Access to cancer screening in people with learning disabilities in the UK: cohort study in the health improvement network, a primary care research database. PLoS One. 2012;7(8):e43841.

39. Ouellette-Kuntz H, Cobigo V, Balogh R, Wilton A, Lunsky Y. The uptake of secondary prevention by adults with intellectual and developmental disabilities. J Appl Res Intellect Disabil. 2015;28(1):43-54.

40. Palmer LD, Heung T, Corral M, Boot E, Brooks SG, Bassett AS. Sexual knowledge and behaviour in 22q11.2 deletion syndrome, a complex care condition. J Appl Res Intellect Disabil. 2022;35(4):966-75.

41. Parish SL, Saville AW, Taylor SJ. Women With Cognitive Limitations Living in the Community: Evidence of Disability-Based Disparities in Health Care. Mental Retardation. 2006;44(4):249-59.

42. Parish SL, Moss K, Richman EL. Perspectives on health care of adults with developmental disabilities. Intellect Dev Disabil. 2008;46(6):411-26.

43. Parish SL, Rose RA, Luken K, Swaine JG, O’Hare L. Cancer Screening Knowledge Changes. Research on Social Work Practice. 2011;22(1):43-53.

44. Parish SL, Swaine JG, Luken K, Rose RA, Dababnah S. Cervical and breast cancer-screening knowledge of women with developmental disabilities. Intellect Dev Disabil. 2012;50(2):79-91.

45. Parish SL, Swaine JG, Son E, Luken K. Determinants of cervical cancer screening among women with intellectual disabilities: evidence from medical records. Public Health Rep. 2013;128(6):519-26.

46. Plourde N, Brown HK, Vigod S, Cobigo V. The Association Between Continuity of Primary Care and Preventive Cancer Screening in Women With Intellectual Disability. Am J Intellect Dev Disabil. 2018;123(6):499-513.

47. Rees G. Increasing access to cancer screening programmes. Learning Disability Practice. 2011;14(7):14-9.

48. Reichard A, Stolzle H, Fox MH. Health disparities among adults with physical disabilities or cognitive limitations compared to individuals with no disabilities in the United States. Disabil Health J. 2011;4(2):59-67.

49. Reichard A, Fox MH. Using population-based data to examine preventive services by disability type among dually eligible (Medicare/Medicaid) adults. Disabil Health J. 2013;6(2):75-86.

50. Reynolds F, Stanistreet D, Elton P. Women with learning disabilities and access to cervical screening: retrospective cohort study using case control methods. BMC Public Health. 2008;8:30.

51. Shin DW, Lee JW, Jung JH, Han K, Kim SY, Choi KS, et al. Disparities in Cervical Cancer Screening Among Women With Disabilities: A National Database Study in South Korea. J Clin Oncol. 2018;36(27):2778-86.

52. Smith AJB, Applebaum J, Tanner EJ, Capone GT. Gynecologic Care in Women With Down Syndrome: Findings From a National Registry. Obstet Gynecol. 2020;136(3):518-23.

53. Son E, Parish SL, Swaine JG, Luken K. Accuracy of self-reported cervical and breast cancer screening by women with intellectual disability. Am J Intellect Dev Disabil. 2013;118(4):327-36.

54. Steele CB, Townsend JS, Courtney-Long EA, Young M. Prevalence of Cancer Screening Among Adults With Disabilities, United States, 2013. Prev Chronic Dis. 2017;14:E09.

55. Stein K, Allen N. Cross sectional survey of cervical cancer screening in women with learning disability. BMJ. 1999;318(7184):641.

56. Stein K. Caring for people with learning disability: a survey of general practitioners’ attitudes in Southampton and South-west Hampshire. British Journal of Learning Disabilities. 2000;28:9-15.

57. Swaine JG, Dababnah S, Parish SL, Luken K. Family caregivers' perspectives on barriers and facilitators of cervical and breast cancer screening for women with intellectual disability. Intellect Dev Disabil. 2013;51(1):62-73.

58. Swaine JG, Parish SL, Luken K, Son E, Dickens P. Test of an intervention to improve knowledge of women with intellectual disabilities about cervical and breast cancer screening. J Intellect Disabil Res. 2014;58(7):651-63.

59. Sykes K, McGeechan GJ, Crawford H, Giles EL. Factor influencing women with learning disabilities deciding to, and accessing, cervical and breast cancer screening: Findings from a Q methodology study of women with learning disabilities, family and paid carers. Eur J Cancer Care (Engl). 2022;31(6):e13702.

60. Wicks S. Assessing the health screening choices of women with learning disabilities. Learning Disability Practice. 2007;10(9).

61. Wood R, Douglas M. Cervical screening for women with learning disability: current practice and attitudes within primary care in Edinburgh. British Journal of Learning Disabilities. 2007;35(2):84-92.

62. Xu X, McDermott SW, Mann JR, Hardin JW, Deroche CB, Carroll DD, et al. A longitudinal assessment of adherence to breast and cervical cancer screening recommendations among women with and without intellectual disability. Prev Med. 2017;100:167-72.

63. Yen SM, Kung PT, Tsai WC. The characteristics and relevant factors of Pap smear test use for women with intellectual disabilities in Taiwan. BMC Health Serv Res. 2014;14:240.
